# A Novel Module Based Method of Teaching Electrocardiogram Interpretation for Emergency Medicine Residents

**DOI:** 10.21980/J8Z06J

**Published:** 2022-10-15

**Authors:** Alexandra S Koutsoubis, Emily Fishbein, Megan Stobart-Gallagher, Behzad B Pavri, Jennifer White

**Affiliations:** * Sydney Kimmel Medical College, Philadelphia, PA; ^ Thomas Jefferson University Hospital, Department of Emergency Medicine, Philadelphia, PA; † Thomas Jefferson University Hospital, Department of Medicine, Division of Cardiology, Philadelphia, PA

## Abstract

**Audience:**

This online learning module is designed for PGY 1–3 emergency medicine (EM) residents.

**Introduction:**

Interpretation of the 12-lead electrocardiogram (ECG) is an essential skill for EM residents. The traditional approach to ECG interpretation in medical school is primarily didactic, teaching: “rate, rhythm, axis,” etc. Throughout residency, EM residents continue to receive lectures and practical ECG teaching to independently interpret ECGs with accuracy and efficiency. In addition to basic rhythm interpretation, physicians must be able to identify cardiac ischemia, abnormal rhythms, and subtle ECG findings that could herald sudden death.^1^ Life-threatening diagnoses such as digitalis toxicity or hyperkalemia can be made promptly through ECG evaluation and catastrophic if missed. If correctly diagnosed through ECG, many channelopathies can be treated and cardiac events can be prevented.^2^,^3^ Lecture-based learning is a necessary part of medical education, but there is a need to supplement the traditional teaching approach with online learning modules. Online learning modules provide learners with an accessible and efficient tool that allows them to improve their ECG skills on their own time.

**Educational Objectives:**

After completion of the module learners should be able to: 1) correctly recognize and identify ECG abnormalities including but not limited to abnormal or absent P waves, widened QRS intervals, ST elevations, abnormal QT intervals, and dysrhythmias that can lead to sudden cardiac death; and 2) synthesize findings into a succinct but accurate interpretation of the ECG findings.

**Educational Methods:**

An online module was developed using Articulate 360 and was implemented with EM residents. The module covers common ECG findings seen in the emergency department including ischemia, atrioventricular blocks, and bundle branch blocks. The module uniquely emphasizes ECG findings of arrythmias that could lead to sudden cardiac death and highlights that diagnosing sudden cardiac death syndromes relies on both clinical presentation and specific ECG findings. Online modules have proven to be as effective as lecture-based learning at improving ECG interpretation among healthcare professionals and are convenient and easily accessible to the busy EM resident.^4^,^5^ Additionally, the module is self-paced, can be completed at any time, and includes elements of active learning by incorporating knowledge checks throughout. This allows learners in real time to see where individualized improvement is needed. The ease of embedment of self-paced questions into the module is one of the salient reasons why module-based learning can be superior to lecture-based learning. This allows for real time retrieval practice, feedback, and repetition, all of which can be powerful and effective tools for learning.^6^

**Research Methods:**

This module was offered at a single academic institution with a 3-year residency program. The investigation was reviewed and approved for exemption by the Institutional Review Board of Sydney Kimmel Medical College. The module was evaluated using survey data; before the module was disseminated, residents were given a pre-module survey. The survey was used to evaluate the methods residents used to interpret ECGs prior to completion of the module and to evaluate their baseline confidence in ECG interpretation. The residents were then given access to the module and had two weeks to complete it. After the two-week period, the post-module survey was used to evaluate resident satisfaction with the delivery of the module, the methods residents used to interpret ECGs after the module, and resident confidence in ECG interpretation. The objective efficacy of the educational content in the module was assessed using a pre- and post-module assessment. The assessments consisted of 15 ECGs.

Residents were asked to provide a one-sentence interpretation for each of the 15 ECGs and the final answers were based on interpretation by an electrophysiologist.

**Results:**

A group of 37 EM residents had two weeks to complete the module between pre- and post-tests. There was an 18.2% absolute increase in the mean percent correct after the module, a 42.5% relative increase from pre-test (t= −8.0, *p* < 0.001). Subjective data demonstrated that after completing the module, residents utilized the novel approach, were more confident in interpreting ECGs, and would use the module as a resource in the future.

**Discussion:**

Most participants were not confident in their ability to interpret an ECG prior to completing the module, despite most of the participants having ECG training in the six months prior to the study. Almost all the participants reported using “rate, rhythm, axis” as their method of ECG interpretation. Even with recent training, and an understanding of “rate, rhythm, axis,” there was a very low accuracy on the pre-test and lack of perceived baseline confidence in this skill. These findings highlight the need for a concise, effective supplemental ECG tool that can be incorporated into residency program curricula.

The online learning module was effective at increasing confidence of ECG interpretation skills in residents as well as increasing accuracy of interpretation. Overall, participants were satisfied with the module as a resource for practicing their ECG interpretation, and most participants reported that they would use the module in the future as a reference. Implementation of the module as an additional resource in resident education is very simple. It can be accessed through any device that has internet and can be completed in a short period of time. Additionally, most experienced ECG readers will speak about “pattern recognition” as an important tool in ECG interpretation. This ability goes above and beyond the “rate, rhythm, axis” approach, but is acquired over time, often after many years of ECG interpretation. It is possible that the modular method may accelerate such pattern recognition abilities.

**Topics:**

Electrocardiogram, online module, sudden cardiac death, ischemia.

## USER GUIDE

**Table:** 

**List of Resources:**	
Abstract	15
User Guide	18
Small Groups Learning Materials	21
Appendix A: Pre-Module Survey and Pre-Test	21
Appendix B: Pre-Test Answers	39
Appendix C: Link to Online Module	40
Appendix D: Post-Test and Post-Module Survey	41
Appendix E: Post-Test Answers	60

**Learner Audience:**

EM Residents (Interns, junior and senior)

**Time Required for Implementation:**

30–45 minutes for the pre-test, 40 minutes for the module, 30–45 minutes for the post-test

**Recommended Number of Learners per Instructor:**

This is an asynchronous module, not requiring moderation in real-time.

**Topics:**

Electrocardiogram, online module, sudden cardiac death, ischemia.

**Objectives:**

By the end of this module, learners should be able to:

1. Correctly recognize and identify ECG abnormalities including but not limited to abnormal or absent P waves, widened QRS intervals, ST elevations, abnormal QT intervals, arrythmias that can lead to sudden cardiac death.
2. Synthesize findings into an accurate one-line interpretation of the ECG findings.

### Linked objectives and methods

An online module was chosen due to its accessibility and efficiency for the EM residents. The module is self-paced and can be completed in one hour or less. This module was created using Articulate Risa 360. Risa 360 is a web-based course builder that requires a purchased subscription to create courses. Courses created on this e-learning tool can be accessed for free without an account or subscription through a direct web-link. https://rise.articulate.com/share/YN7pQLkc3HJeTHSnLqCLK-RRQLk8j3Uj

The module summarizes basic ECG knowledge and reviews electrical abnormalities seen on ECG, while also teaching a novel approach to synthesizing information into an accurate interpretation. The module guides learners through the following steps: 1) Is it normal sinus rhythm? 2) Is the QRS wide or narrow? 3) Is there myocardial ischemia/injury? 4) Does this ECG herald sudden death? This approach allows learners to synthesize information gathered from the ECG into a meaningful interpretation. Seasoned providers use pattern recognition to interpret ECGs, which is difficult to teach; therefore, most lectures and modules use the traditional “rate, rhythm, axis” approach to teach residents. This module utilizes a unique approach that emphasizes pattern recognition throughout the learning process. The module also includes frequent knowledge checks that allow the learners to self-evaluate their progress. These knowledge checks provide learners the opportunity to identify ECG abnormalities and integrate the findings to determine an interpretation of the ECG as a whole.

Online learning modules are effective at improving ECG interpretation among healthcare professionals.^4^,^5^ Also, e-learning modules have been shown to be as effective as lecture-based learning in improving ECG interpretation by residents.^5^ Current literature suggests that as newer versions of ECG training modules are developed, an emphasis on diagnosing electrical diseases is needed.^4^

The current module created provides a unique ECG resource that is tailored to be accessible to EM residents. Its application will be used as an additional resource to any ECG curriculum currently being taught at residency programs. ECG modules have also been developed that contain case-based ECG practice, but this module serves as a resource that is a broad review of ECG principles as well as specific ECG abnormalities.^7^ This module is also unique because it teaches learners a systematic approach to ECG interpretation that facilitates the learner to come to a “one line” diagnosis. This module is easily accessible even on personal smartphones and can be completed in under an hour, serving as a resource to residents whenever they need it.

### Small group application exercise (sGAE)

This module can be used alone or in conjunction with pre- and post-module assessments and questionnaires. To complete the module with the pre- and post-module assessments and questionnaires, provide the learners with the pre-module assessment and questionnaire (Appendix A) either in person or remotely via an online platform such as Google Forms. The learners should complete the initial questionnaire which assesses subjective information regarding ECGs. Then provide the learners with the pre-test in which they interpret a set of 15 ECGs and submit a one-line interpretation for each ECG in a closed book fashion. The participants should be scored on their interpretation based on final answers provided by an electrophysiologist. Answers are graded as correct or incorrect. After completion of the pre-test, give the participants access to the online learning module link via email (Appendix C). Instruct the learners to complete the module on their own time. After completion of the module, give the participants the postmodule test and questionnaire (Appendix D) in the same format as the pre-module. The post-module questionnaire subjectively assesses the learner’s opinion about the module and reassesses confidence of ECG interpretation. The post-test consists of another set of 15 ECGs with similar findings and should be scored in the same fashion. Answer keys to the pre- and post-test can also be found below.

### Results and tips for successful implementation

The module and pre- and post-module assessments and surveys were implemented in two ways. A total of 37 residents (first-, second-, and third-year) participated in the study. For half of the participants, the pre-module assessment and survey was introduced during the weekly half-day in-person resident conference. This was a convenient time because most of the residents were available. For the other half of the participants, the implementation of the module was entirely virtual due to COVID-19 precautions. The pre- and post-module assessments and surveys were implemented using an online platform. Although the introduction to the module and the pre- and postmodule assessment and questionnaires varied from in-person to virtual, the results were similar.

Data collected from the pre-module survey showed that 95% of residents use “rate, rhythm, axis” as their method to interpret ECGs. While most residents use this conventional method, only 30% of participants were “confident” in interpreting ECGs, with 54% being “less confident” and 16% being “not confident.” The pre-module survey showed that 69% of participants had recent ECG training in the form of lecture or module in the six months prior to participating in this study.

After completion of the pre-module assessment and survey, the residents were given access to the module for two weeks and then asked to sit for a post-module assessment and questionnaire (half in person and half virtual). The post-module survey results showed that most participants reported spending between 20 and 40 minutes on the module. Sixty-nine percent of participants found it useful to be able to complete the module on their own time as opposed to gaining the information in normal lecture style. Eighty-eight percent of participants said they would use the module to review ECGs in the future. The survey showed that during the post-test, 66% of participants used the novel approach to interpret the ECGs while 33% did not. Seventy-five percent of residents said they would try to use the novel approach in the future while interpreting ECGs. After the module, 51% reported being confident in interpreting ECGs (up from 30%), 42% reported being less confident (down from 57%), and 7% being not confident (down from 18%).

**Figure 1:**
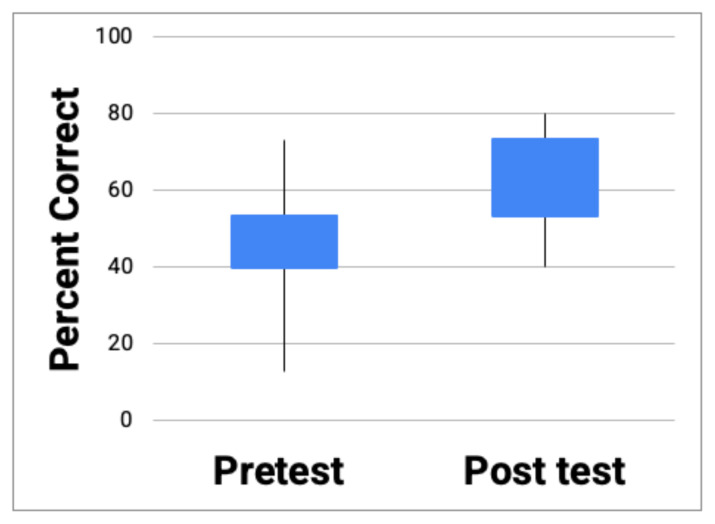
Boxplot showing mean percent correct (42.5%) on the pre-test completed before the module and mean percent correct (61%) on the post-test completed after the module ( t= −8.0, p < 0.001).

The objective data showed that the mean percent correct on the pre-test was 42.8%. The mean percent correct on the post-test, after completing the module, was 61%. There was an 18.2% absolute increase in mean percent correct after the module (a 42.5% relative increase from the pre-test), which was statistically significant (t= −8.0, p < 0.001; Fig. 1).

While the study was implemented in two different fashions (in person vs. virtually) the results did not change. In fact, implementing the module virtually fostered more participation from the residents. Hence, we suggest implementing future studies virtually. The most common suggestion to improve the module was for more examples of various ECG findings and for videos to be embedded throughout the module.

### Learner responsible content (LRC)

This entire module can be done as asynchronous learning.

### Associated content/sections of didactics

Appendix A: Pre-Module Survey and Pre-Test
Appendix B: Pre-Test Answers
Appendix C: Link to Online Module
Appendix D: Post-Test and Post-Module Survey
Appendix E: Post-Test Answers

## SMALL GROUPS LEARNING MATERIALS

### Appendix C Link to Online Module

https://rise.articulate.com/share/YN7pQLkc3HJeTHSnLqCLK-RRQLk8j3Uj
