# Supplementary material for: A Novel Module Based Method of Teaching Electrocardiogram Interpretation for Emergency Medicine Residents
Source: J Educ Teach Emerg Med. 2022 Oct 15;7(4):SG15–60. doi: 10.21980/J8Z06J (PMC10332672; doi:10.21980/J8Z06J)
Supplement: Supplementary file 1 [file JETem-7-4-SG15-AppendixA.docx]

Appendix A:

Pre-Module Survey and Pre-Test

**ECG Pre-Module Survey**

1. Do you currently use “Rate, Rhythm, Axis” when interpreting ECGs
   1. Yes
   2. No
2. If no, please explain your method

1. When was the last time you had a lecture or module on ECG interpretation
   1. Within the last 6 months
   2. Within the last year
   3. Over a year ago
2. How confident are you with interpreting ECGs?
   1. Very confident
   2. Confident
   3. Less confident
   4. Not confident
3. How confident are you in calculating the QTc?
   1. Very confident
   2. Confident
   3. Less confident
   4. Not confident

**ECG Module Pre-Test**

Instructions

Please write your name at the top of your answer sheet. When the timer starts you can open your packet of 15 ECGs. Please write “one liner” interpretation on your answer sheet, as if you were telling the attending what the ECG shows. Keep in mind that there is no partial credit. You have to include all of the abnormalities to get the answer correct. Below is an example of a “correct interpretation”. This example is a STEMI, to get the answer correct you also need the localization. When you are finished, please raise your hand and we will document your time and hand out a quick survey for you to complete.

Example


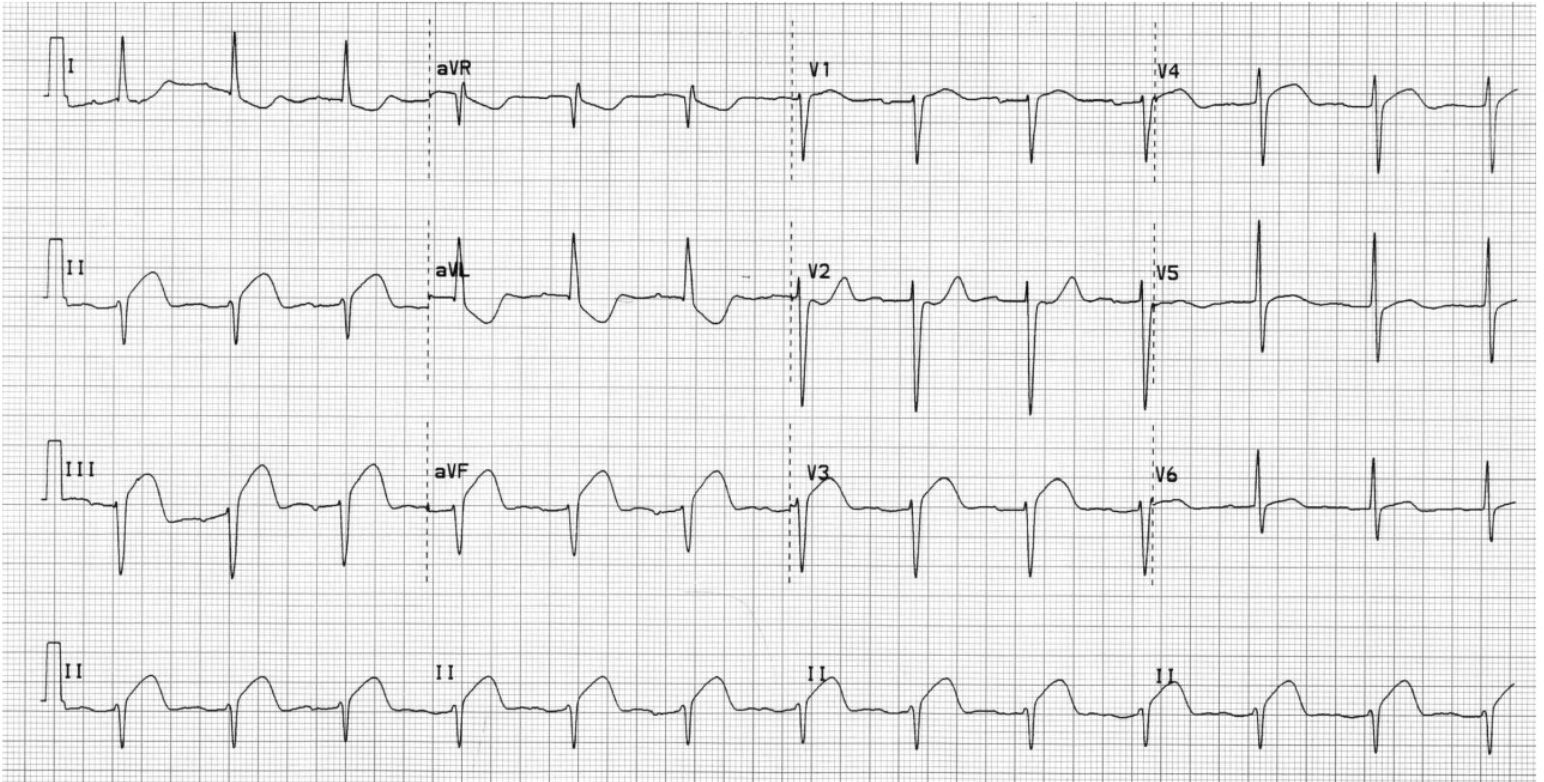


Image retrieved from: [<https://kchemekg.wordpress.com/>] on July 2019. Permission received from Mark Silverberg

**Acceptable response:** Normal sinus rhythm with anterior inferior ischemia OR ST elevations c/w anterior inferior ischemia

**Not acceptable:** Normal sinus rhythm with ischemia/ ST elevations

**Pre-Test Answer Sheet**

Name: _____________________________________________ Resident Class: _______________________

1. ____________________________________________________________________________________
2. ____________________________________________________________________________________
3. ____________________________________________________________________________________
4. ____________________________________________________________________________________
5. ____________________________________________________________________________________
6. ____________________________________________________________________________________
7. ____________________________________________________________________________________
8. ____________________________________________________________________________________
9. ____________________________________________________________________________________
10. ____________________________________________________________________________________
11. ____________________________________________________________________________________
12. ____________________________________________________________________________________
13. ____________________________________________________________________________________
14. ____________________________________________________________________________________
15. ____________________________________________________________________________________

ECG Module Pre-Test: ECG 1


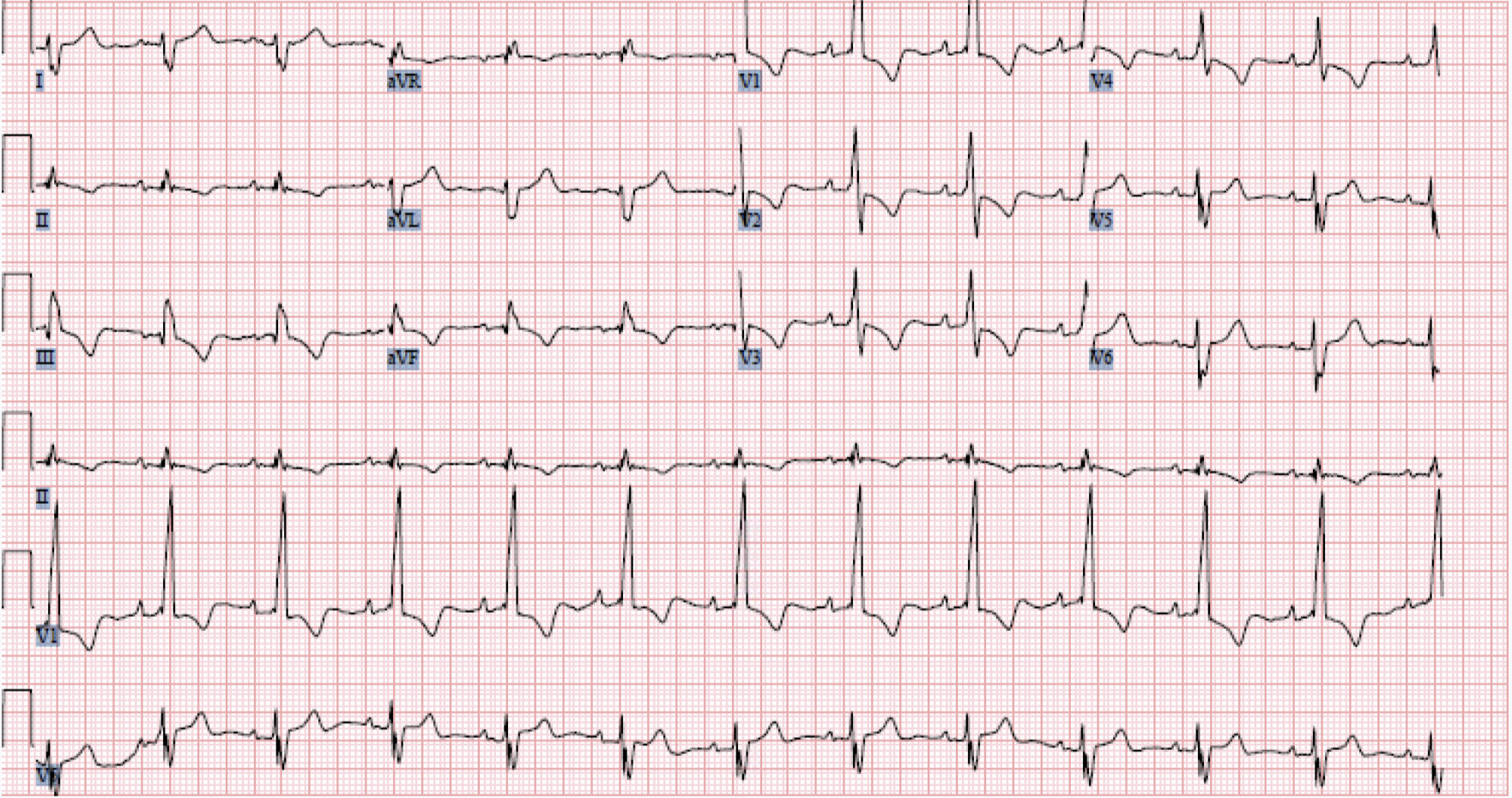


*Image by Dr. Jennifer White, Author Owned*

ECG Module Pre-Test: ECG 2


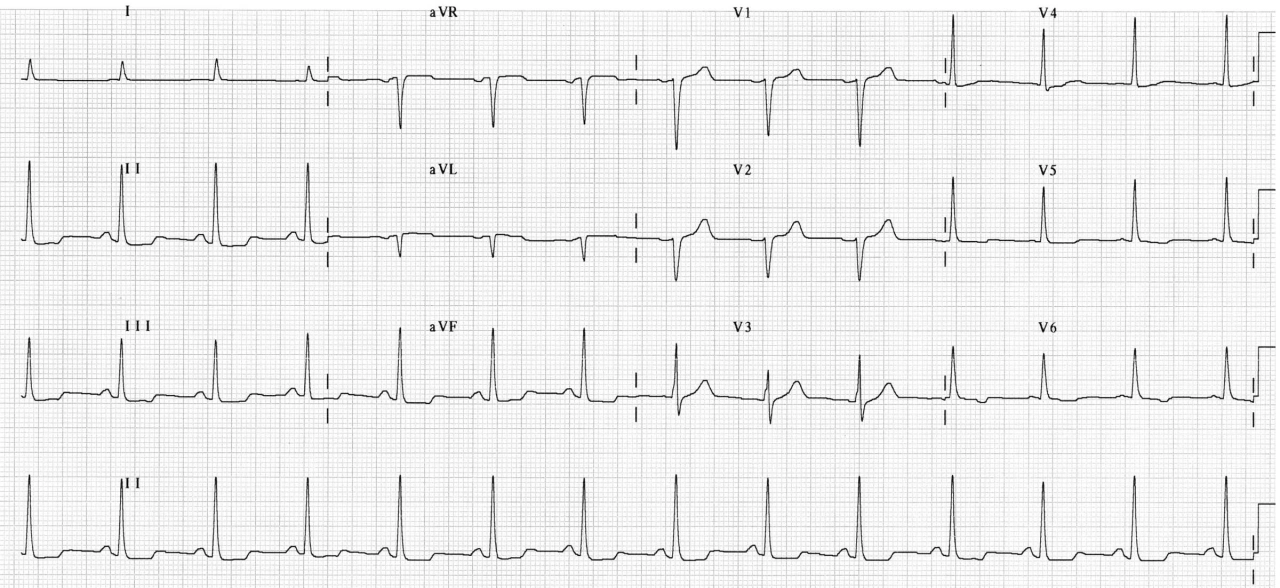


Image retrieved from: [<https://kchemekg.wordpress.com/>] on July 2019. Permission received from Mark Silverberg

ECG Module Pre-Test: ECG 3


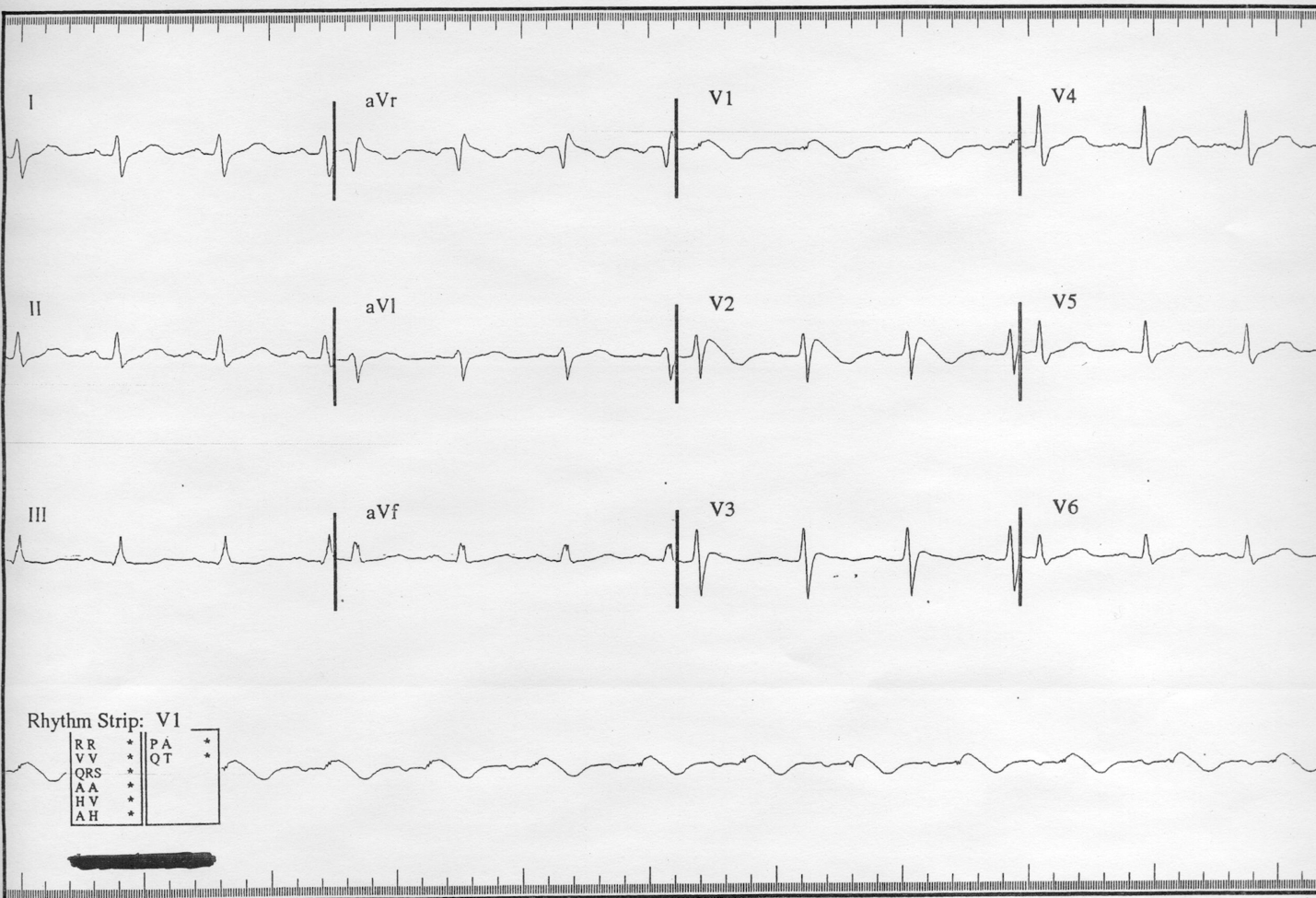


*Image by Dr. Behzad Pavri, Author Owned*

ECG Module Pre-Test: ECG 4


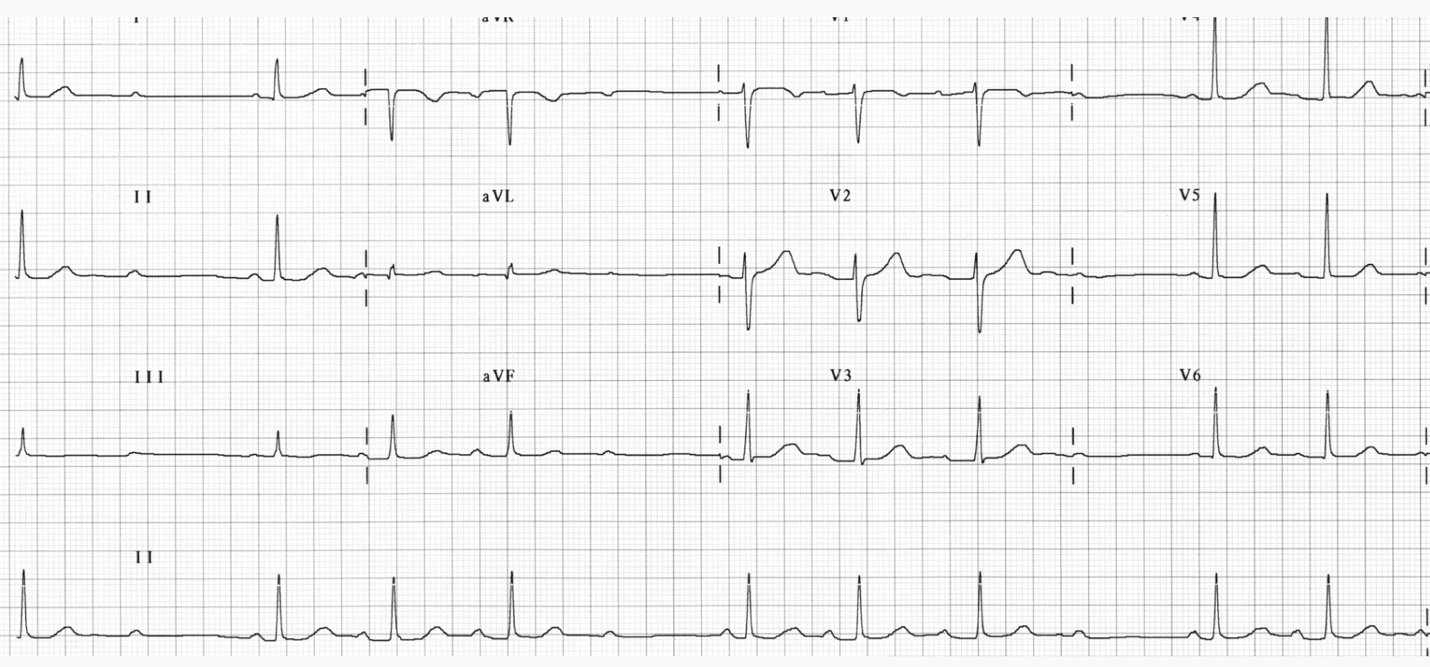


*Image by Dr. Jennifer White, Author Owned*

ECG Module Pre-Test: ECG 5


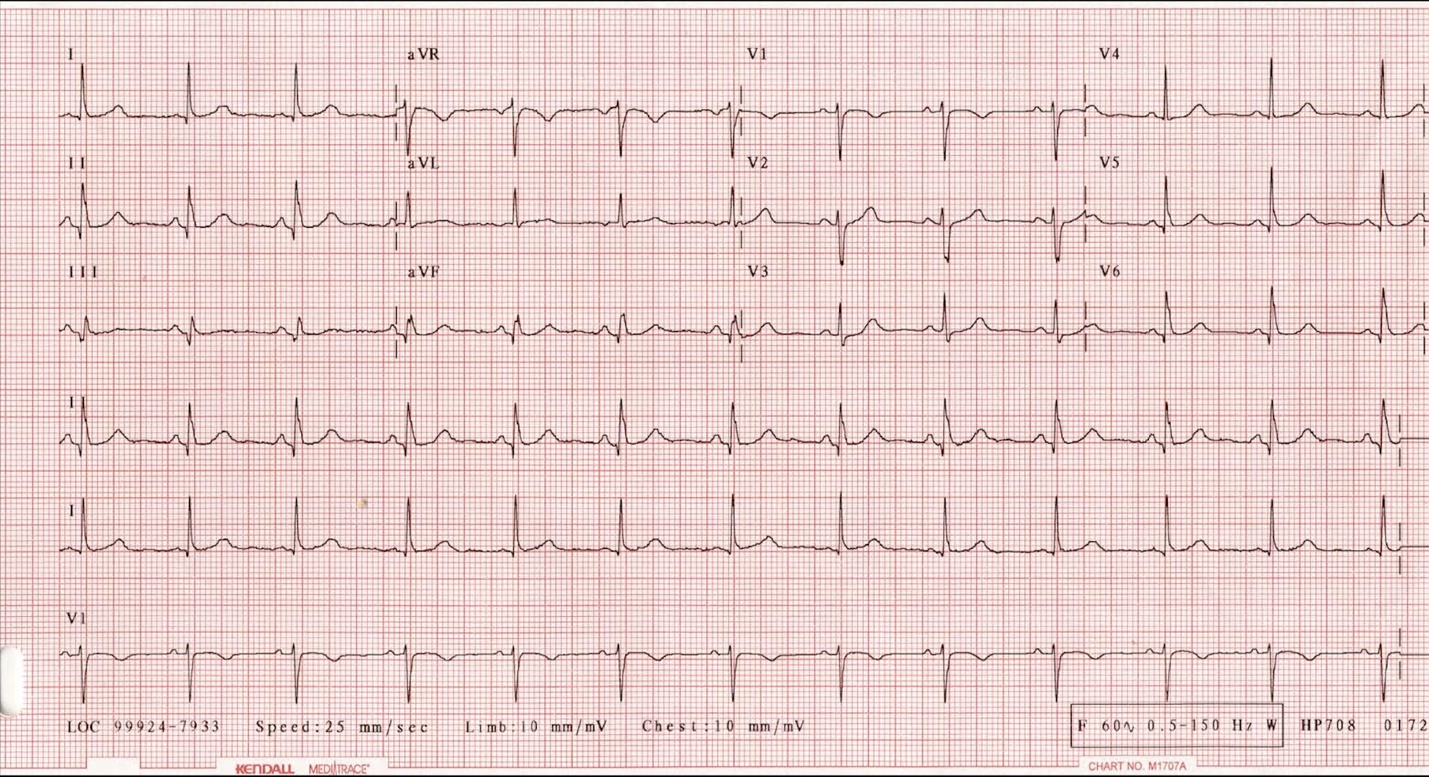


*Image by Dr. Behzad Pavri, Author Owned*

ECG Module Pre-Test: ECG 6


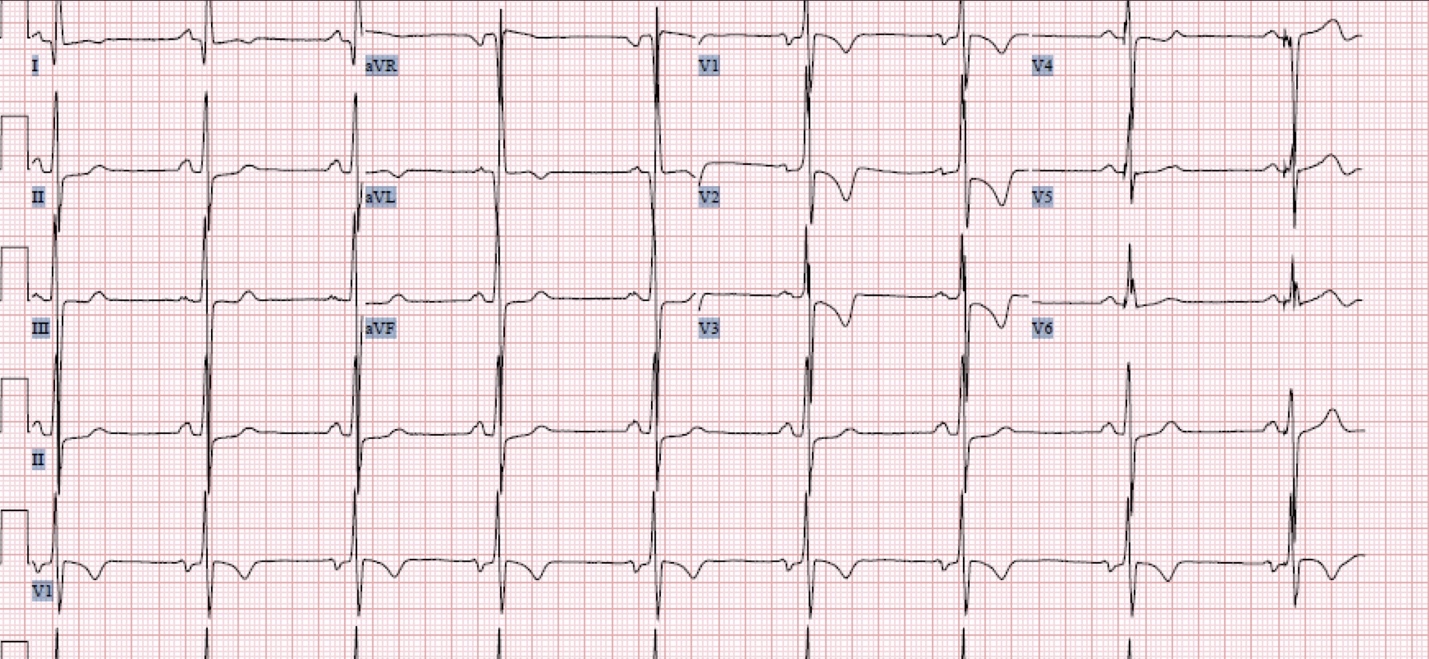


*Image by Dr. Jennifer White, Author Owned*

ECG Module Pre-Test: ECG 7


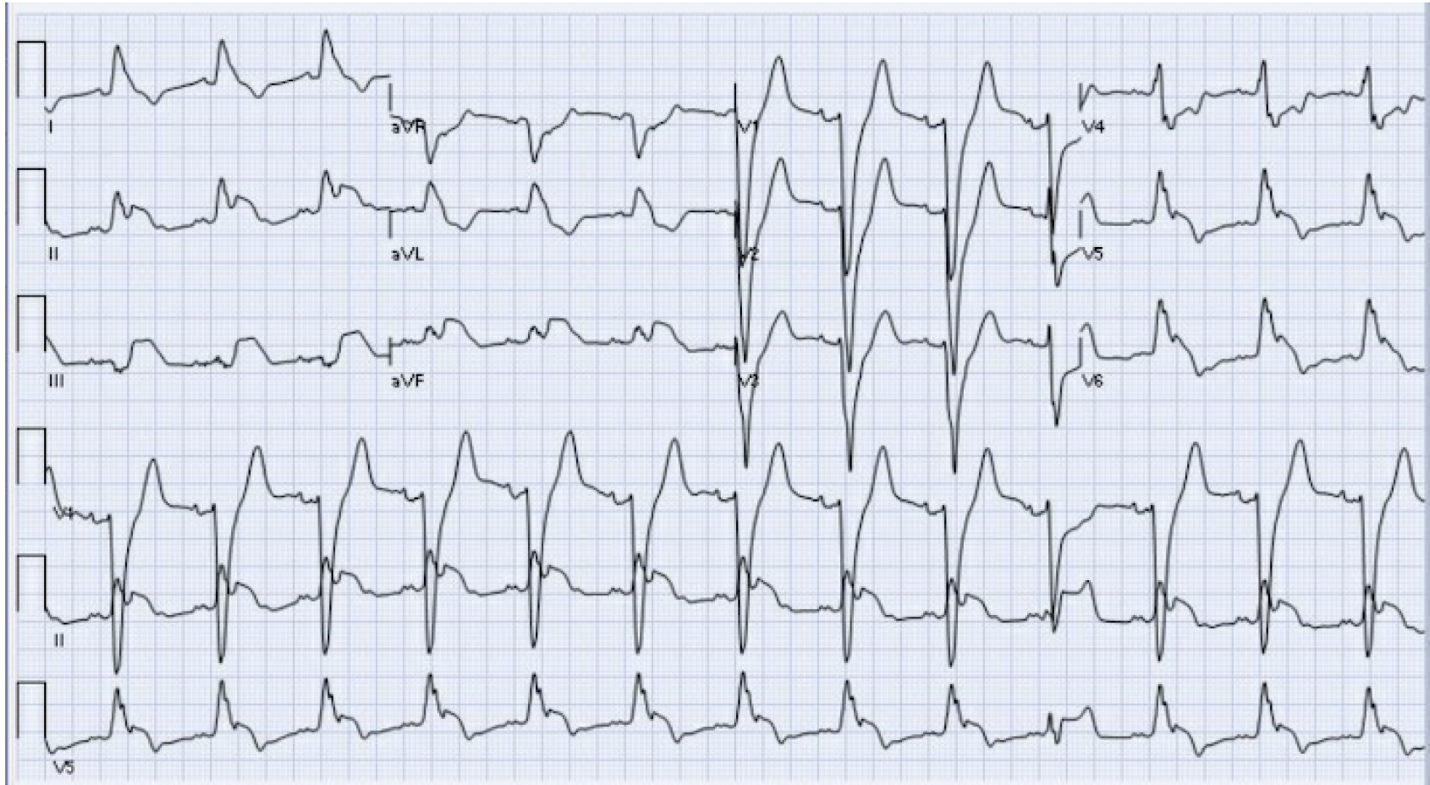


*Image by Dr. Behzad Pavri, Author Owned*

ECG Module Pre-Test: ECG 8


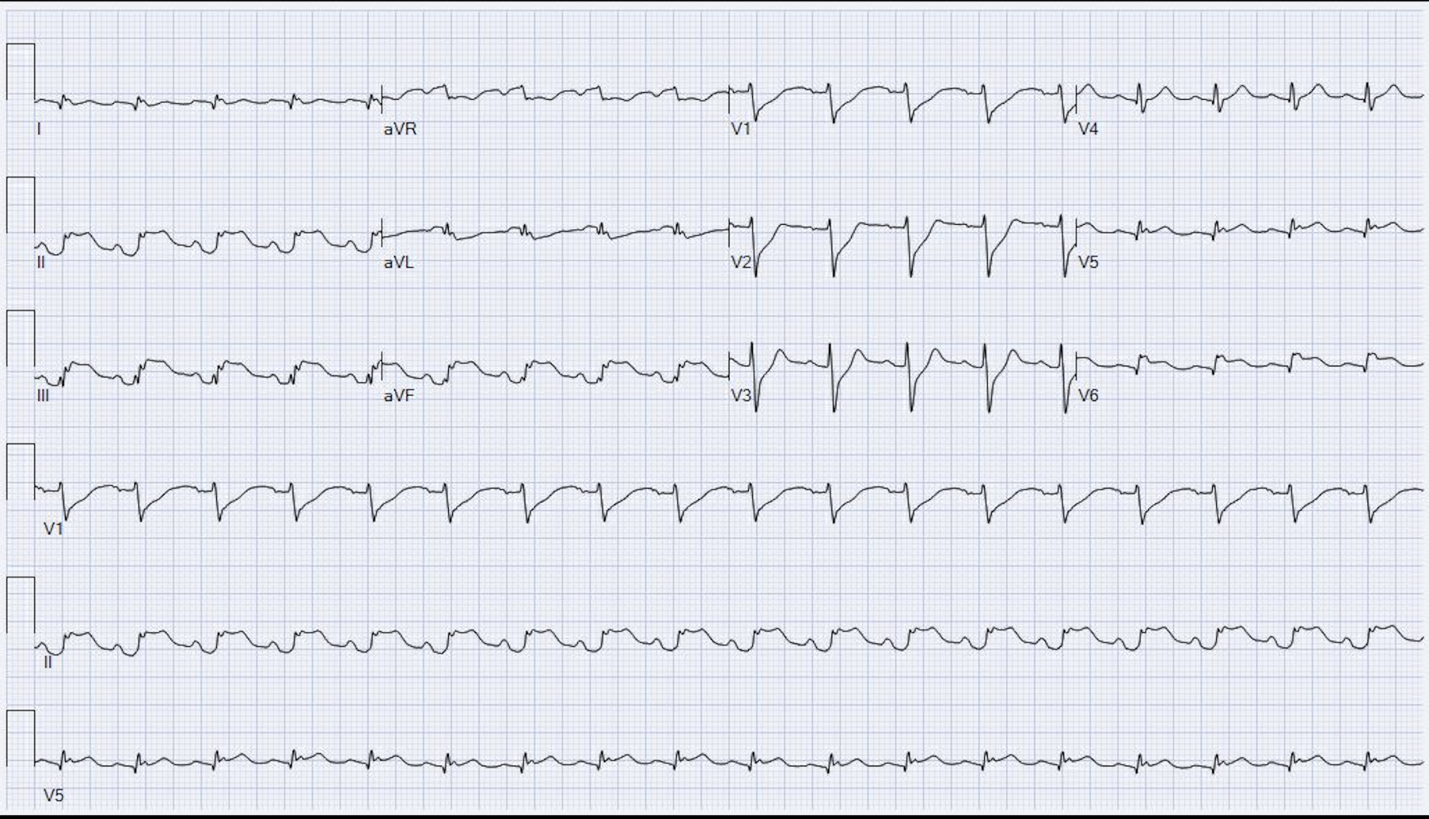


*Image by Dr. Behzad Pavri, Author Owned*

ECG Module Pre-Test: ECG 9


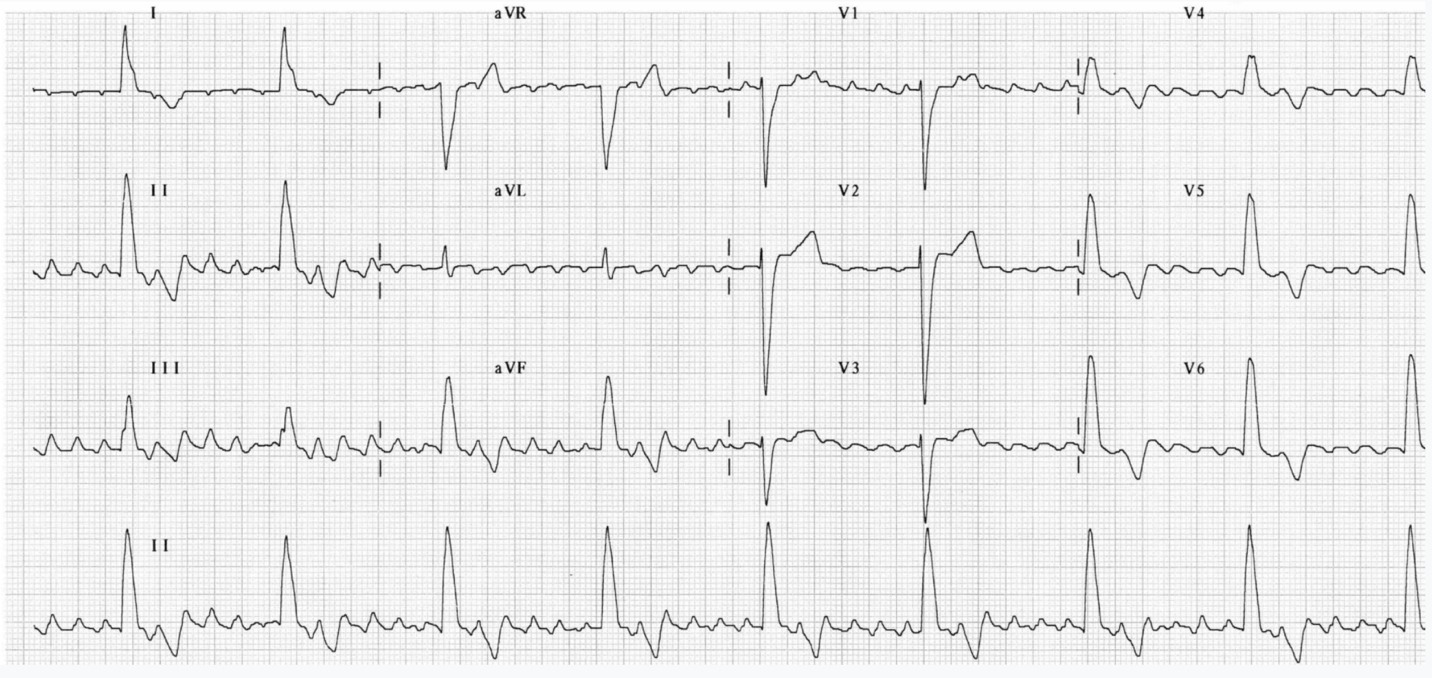


*Image by Dr. Jennifer White, Author Owned*

ECG Module Pre-Test: ECG 10


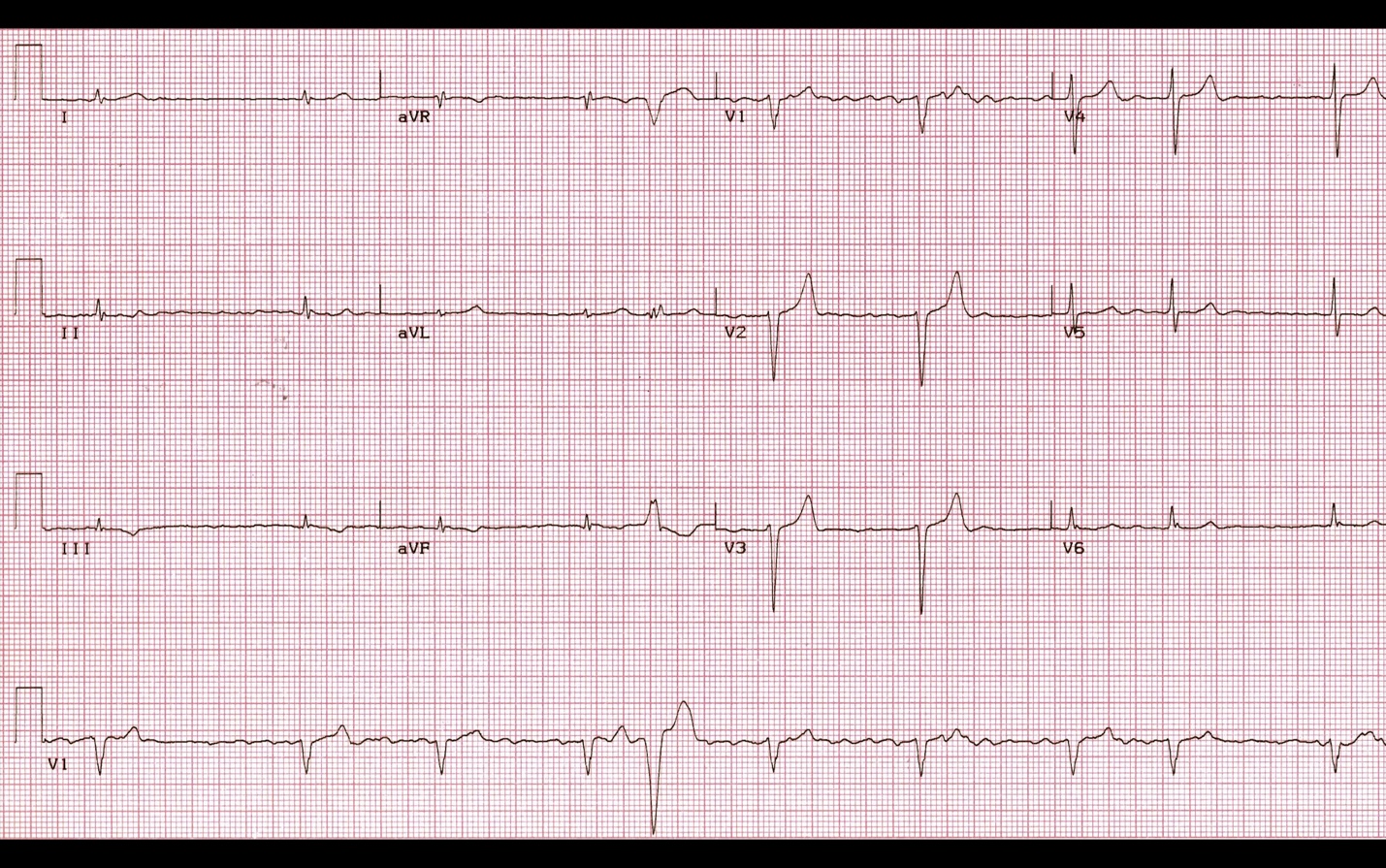


*Image by Dr. Behzad Pavri, Author Owned*

ECG Module Pre-Test: ECG 11


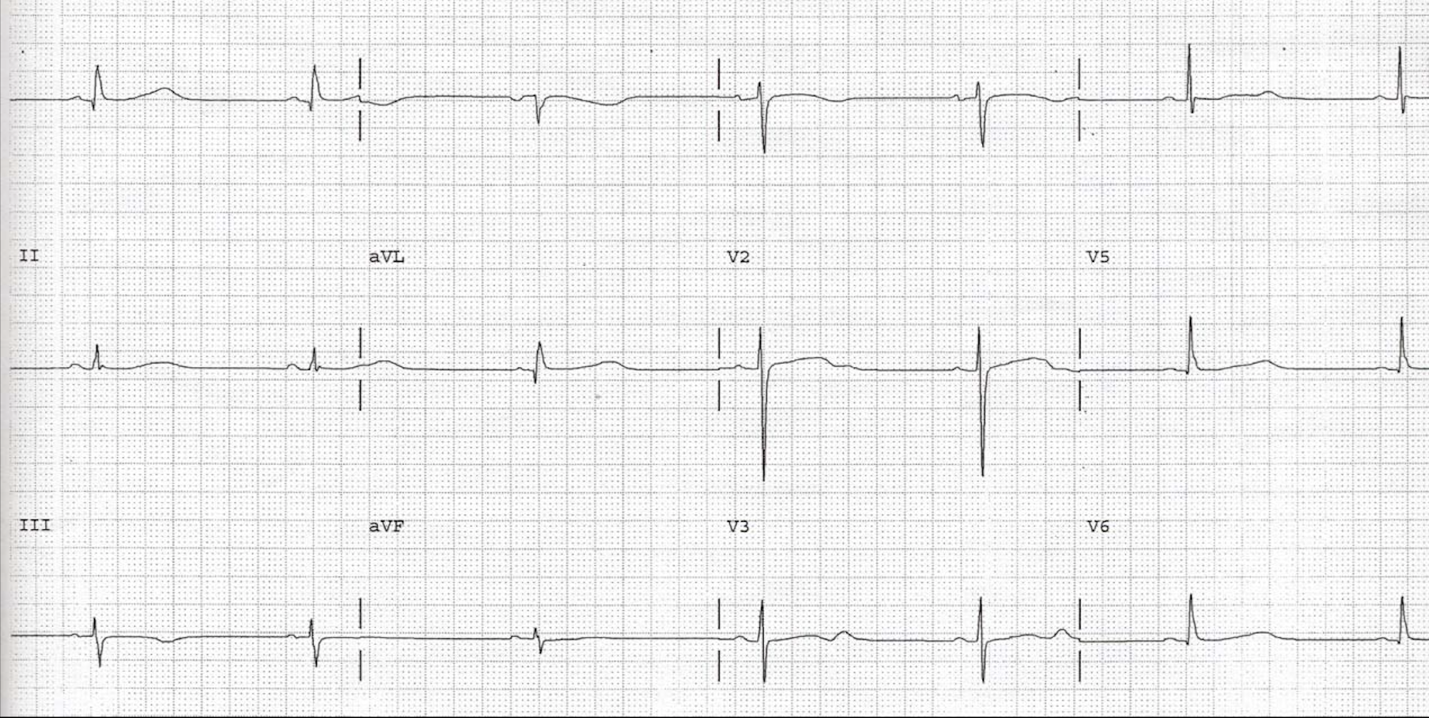


*Image by Dr. Behzad Pavri, Author Owned*

ECG Module Pre-Test: ECG 12


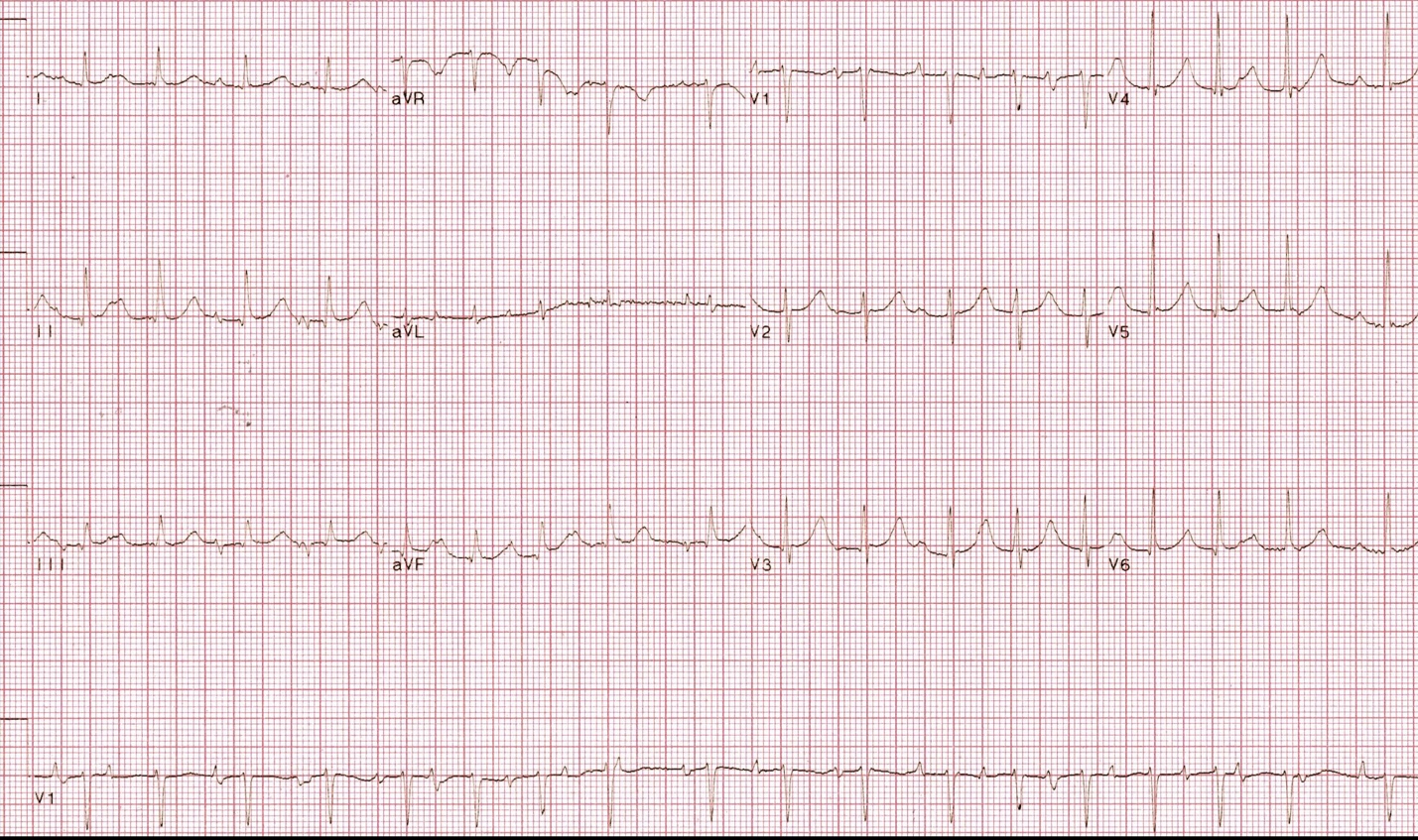


*Image by Dr. Behzad Pavri, Author Owned*

ECG Module Pre-Test: ECG 13


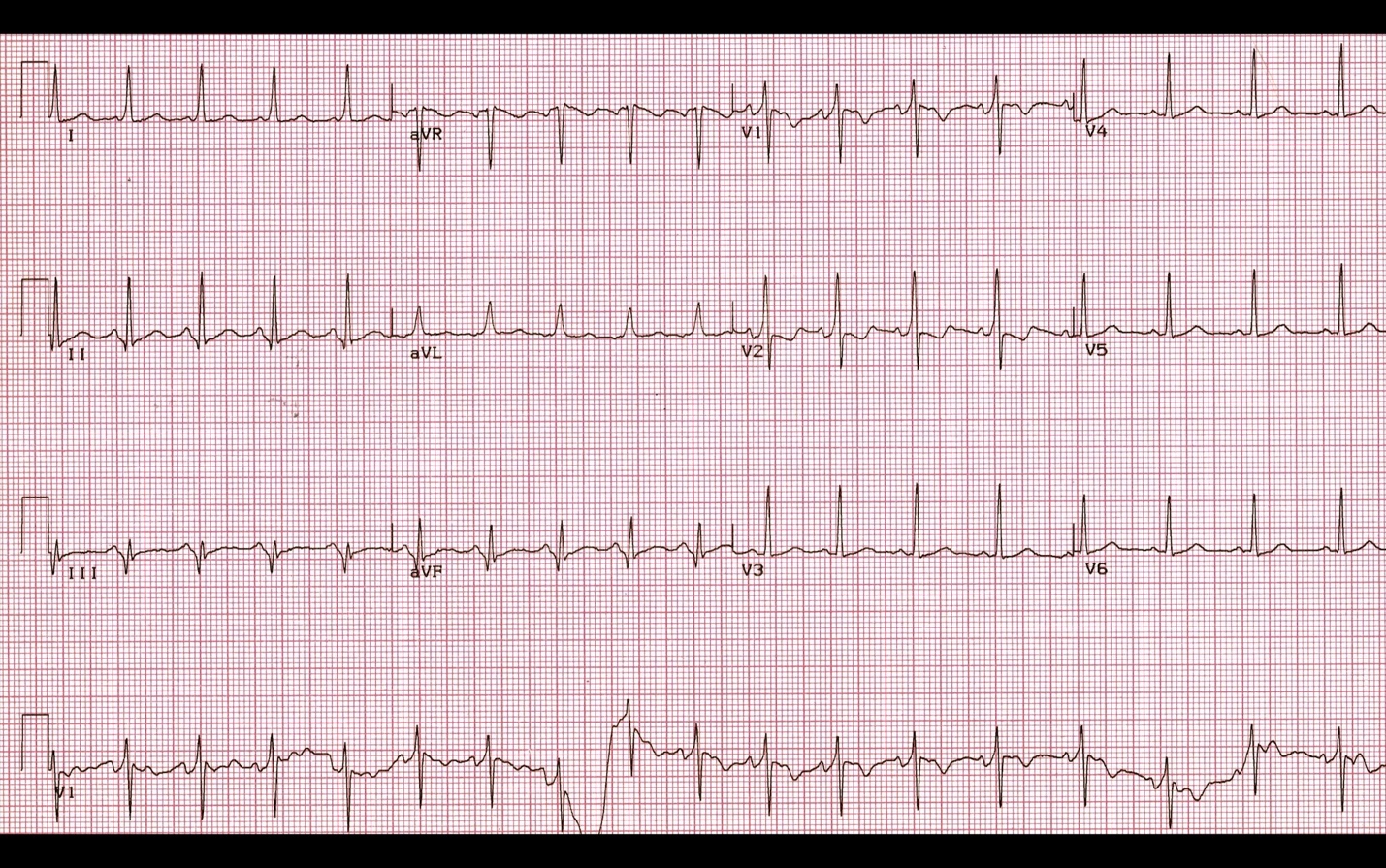


*Image by Dr. Behzad Pavri, Author Owned*

ECG Module Pre-Test: ECG 14


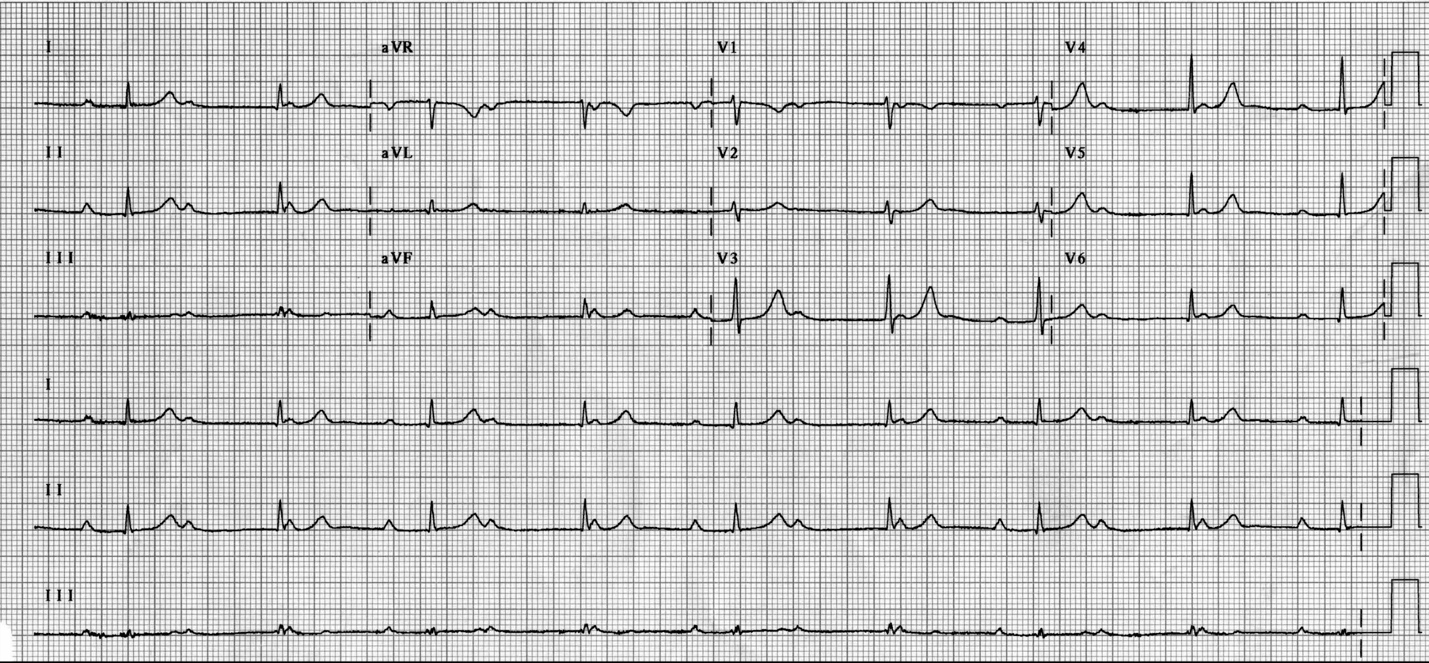


*Image by Dr. Behzad Pavri, Author Owned*

ECG Module Pre-Test: ECG 15


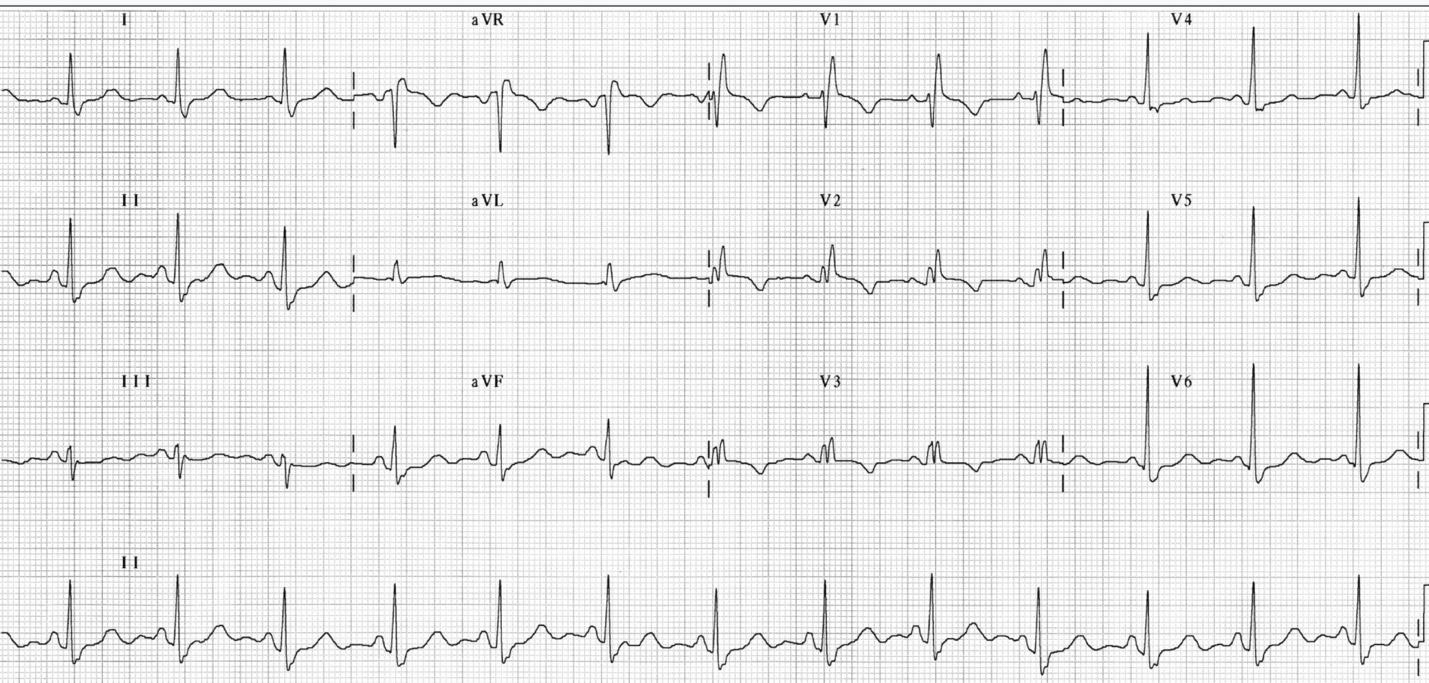


*Image by Dr. Jennifer White, Author Owned*
