# Supplementary material for: A Novel Module Based Method of Teaching Electrocardiogram Interpretation for Emergency Medicine Residents
Source: J Educ Teach Emerg Med. 2022 Oct 15;7(4):SG15–60. doi: 10.21980/J8Z06J (PMC10332672; doi:10.21980/J8Z06J)
Supplement: Supplementary file 2 [file JETem-7-4-SG15-AppendixA1.pptx]

## Slide 1
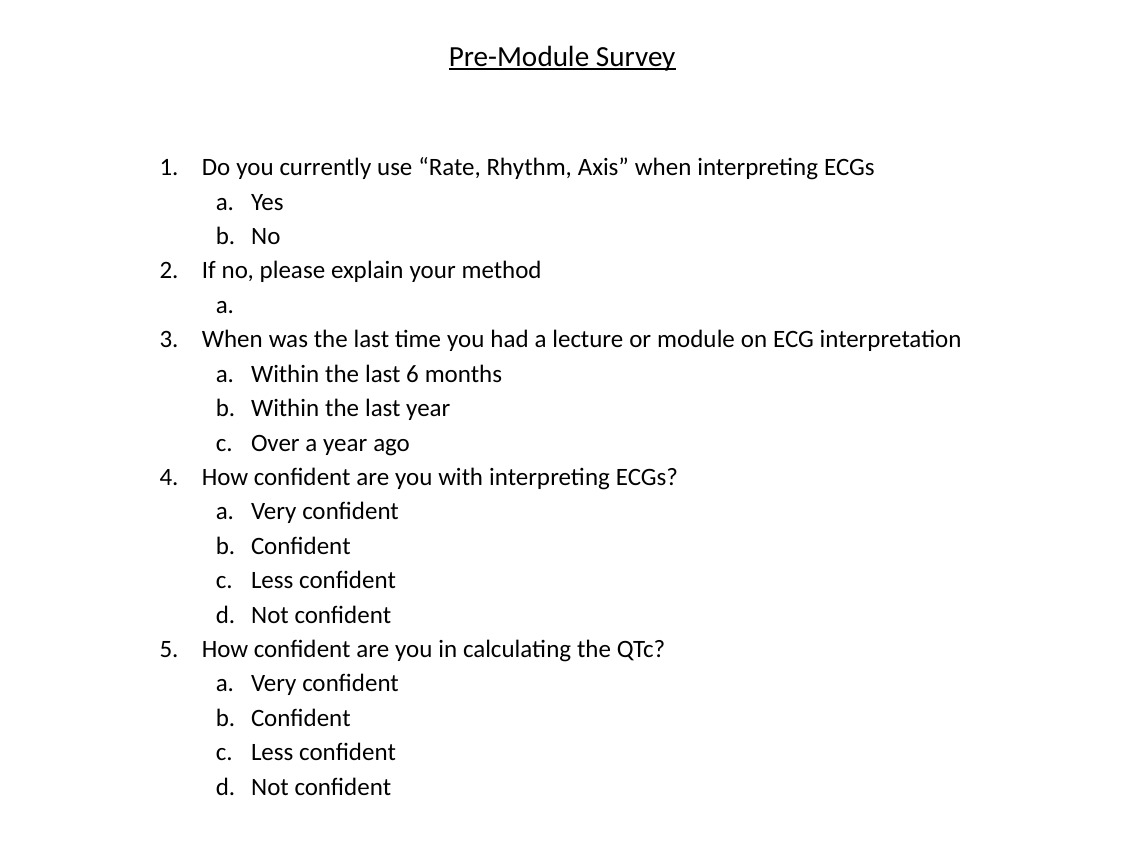

Pre-Module Survey
Do you currently use “Rate, Rhythm, Axis” when interpreting ECGs
Yes
No
If no, please explain your method
When was the last time you had a lecture or module on ECG interpretation
Within the last 6 months
Within the last year
Over a year ago
How confident are you with interpreting ECGs?
Very confident
Confident
Less confident
Not confident
How confident are you in calculating the QTc?
Very confident
Confident
Less confident
Not confident

## Slide 2
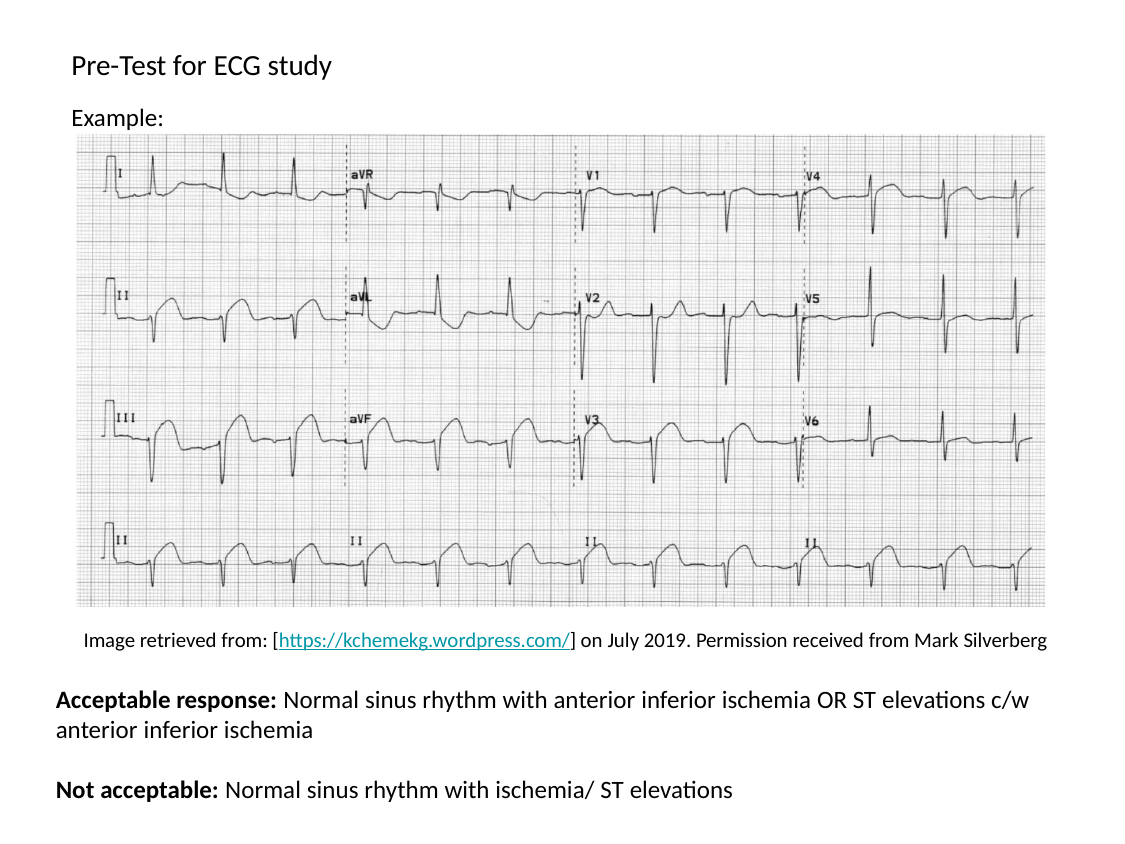

Pre-Test for ECG study
Example:
Image retrieved from: [https://kchemekg.wordpress.com/] on July 2019. Permission received from Mark Silverberg
Acceptable response: Normal sinus rhythm with anterior inferior ischemia OR ST elevations c/w anterior inferior ischemia
Not acceptable: Normal sinus rhythm with ischemia/ ST elevations

## Slide 3
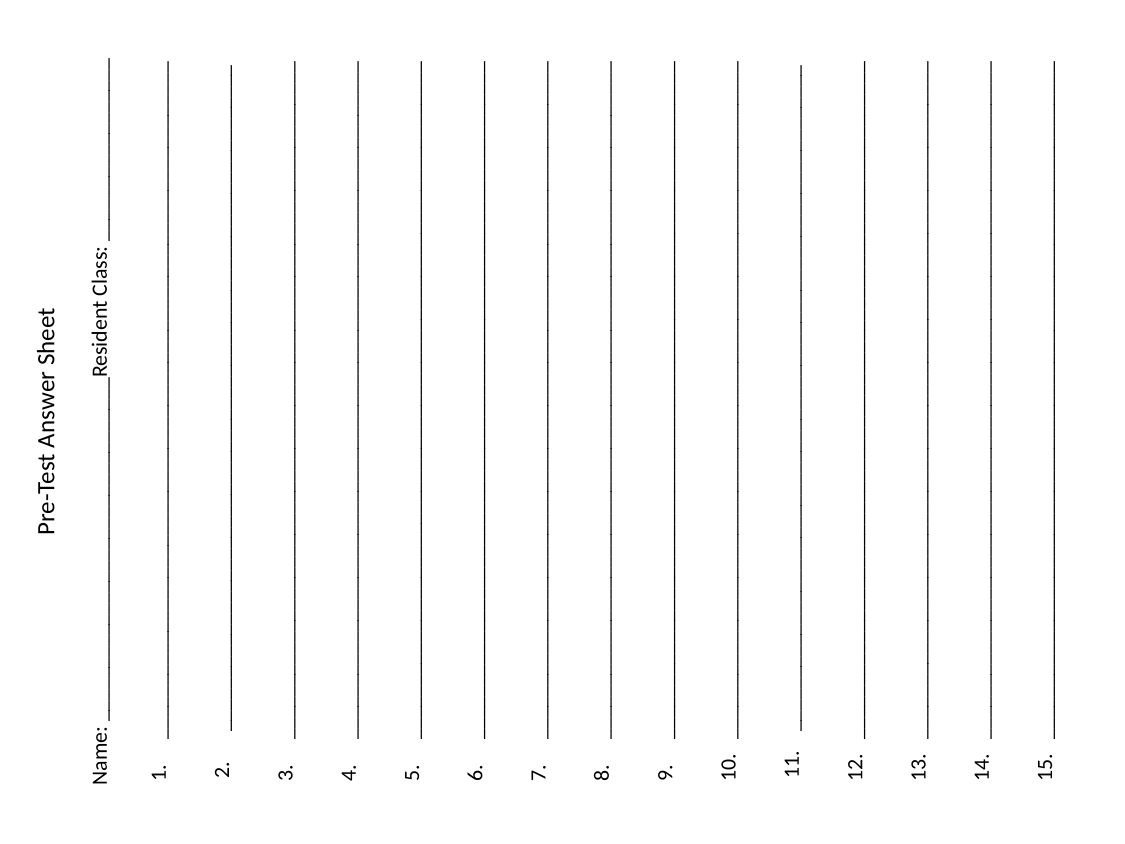

Pre-Test Answer Sheet
Name: ________________________________Resident Class: _________________
_______________________________________________________________
 ______________________________________________________________
_______________________________________________________________
_______________________________________________________________
_______________________________________________________________
_______________________________________________________________
_______________________________________________________________
_______________________________________________________________
_______________________________________________________________
_______________________________________________________________
 ______________________________________________________________
_______________________________________________________________
_______________________________________________________________
_______________________________________________________________
_______________________________________________________________

## Slide 4
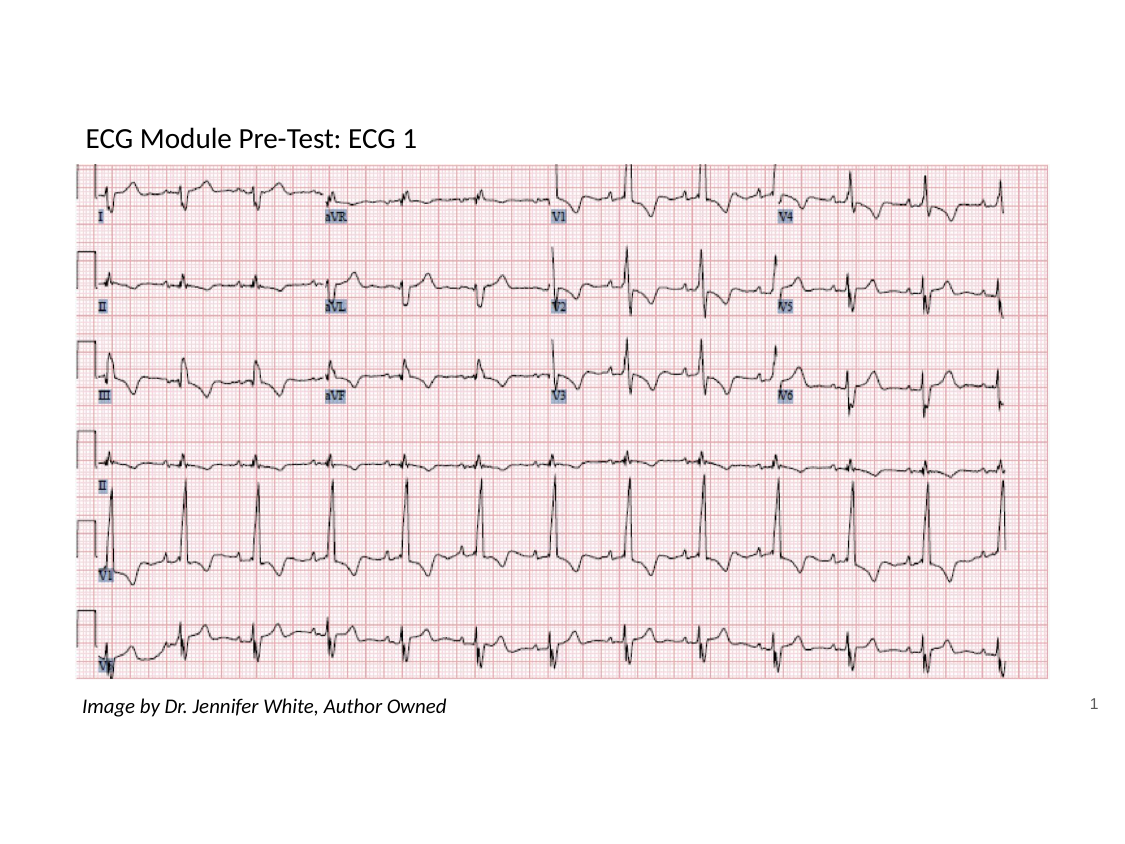

ECG Module Pre-Test: ECG 1
1
Image by Dr. Jennifer White, Author Owned

## Slide 5
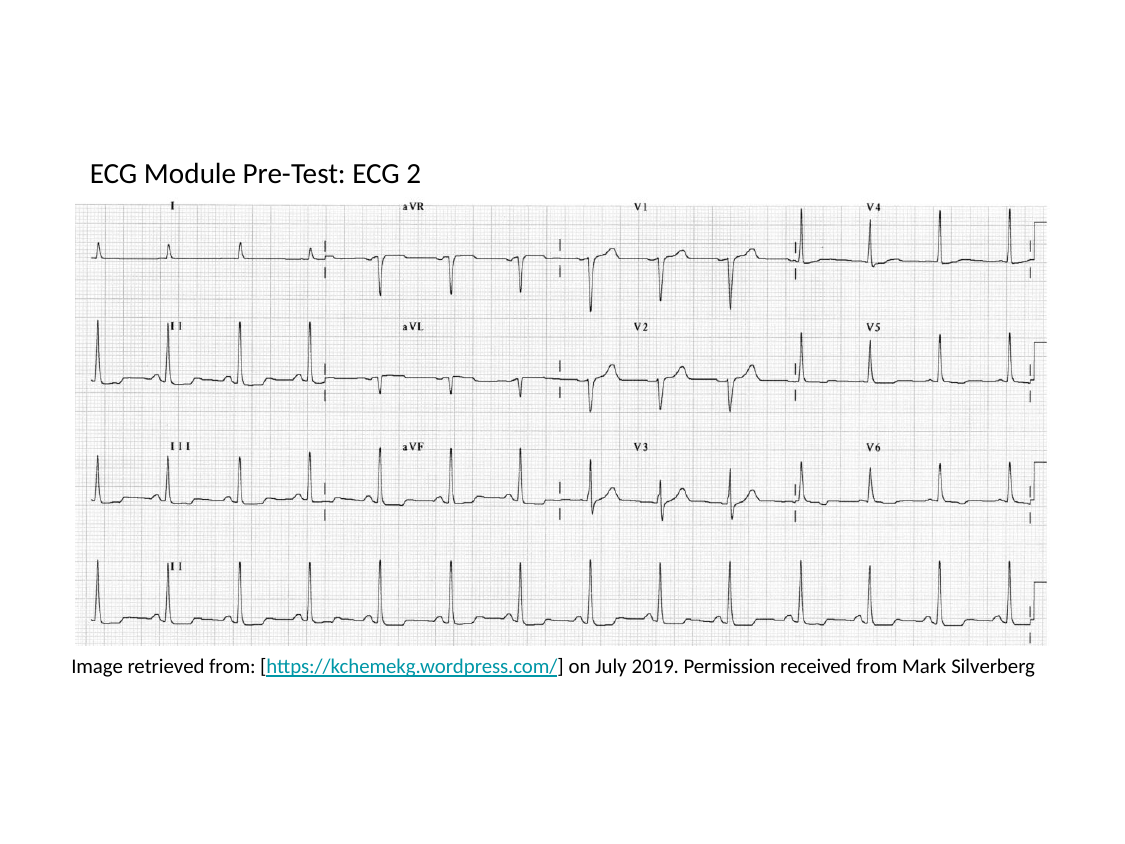

ECG Module Pre-Test: ECG 2
Image retrieved from: [https://kchemekg.wordpress.com/] on July 2019. Permission received from Mark Silverberg

## Slide 6
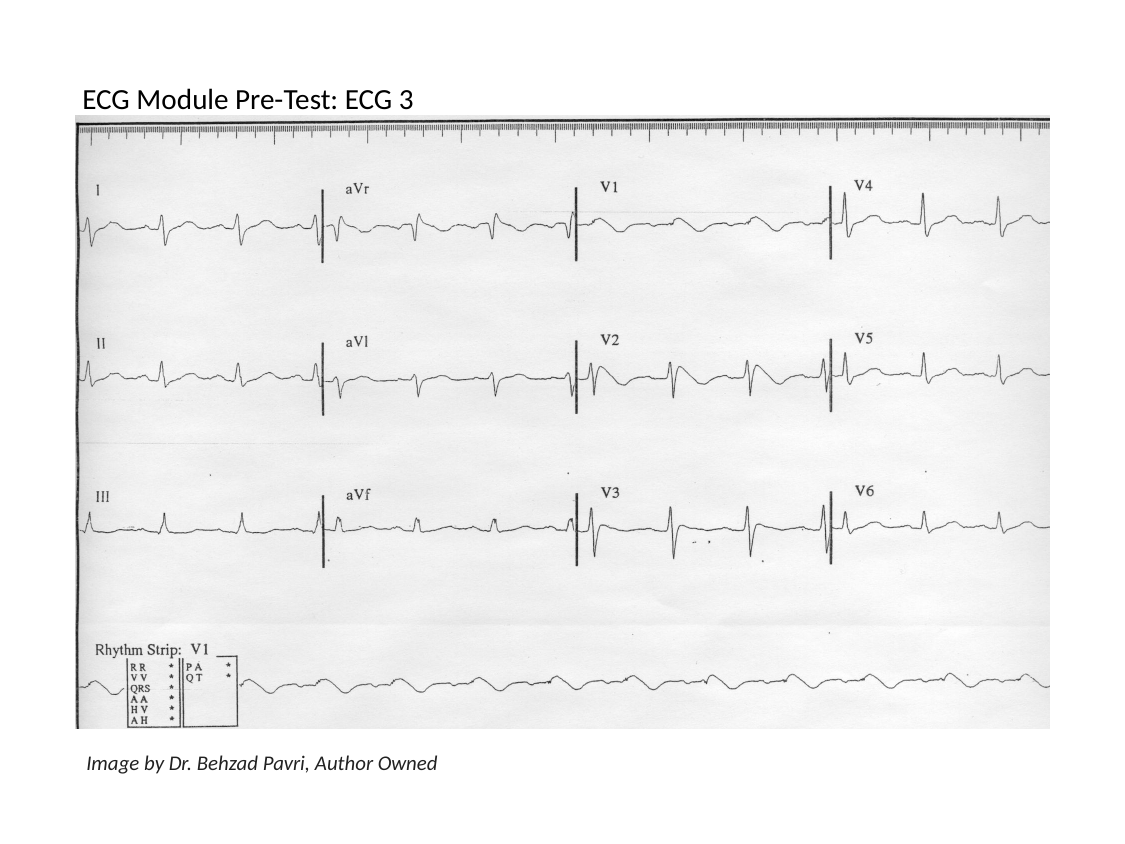

ECG Module Pre-Test: ECG 3
Image by Dr. Behzad Pavri, Author Owned

## Slide 7
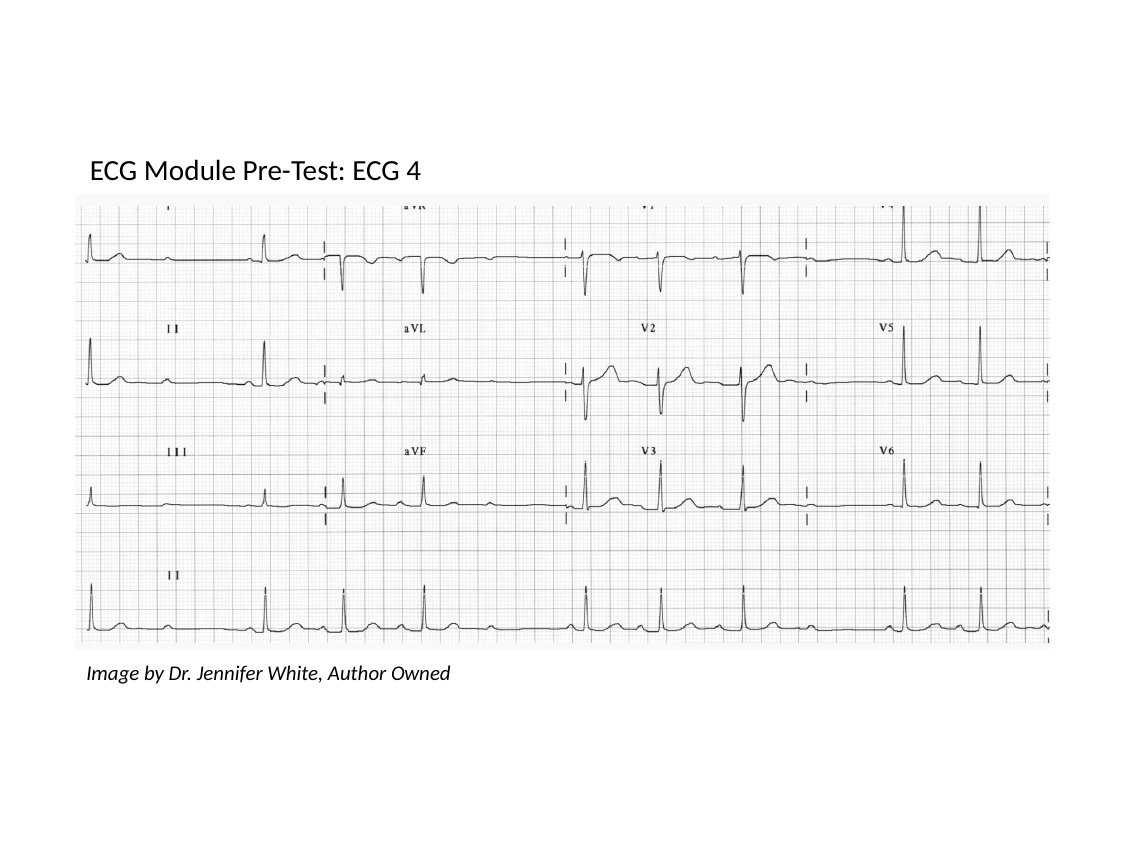

ECG Module Pre-Test: ECG 4
Image by Dr. Jennifer White, Author Owned

## Slide 8
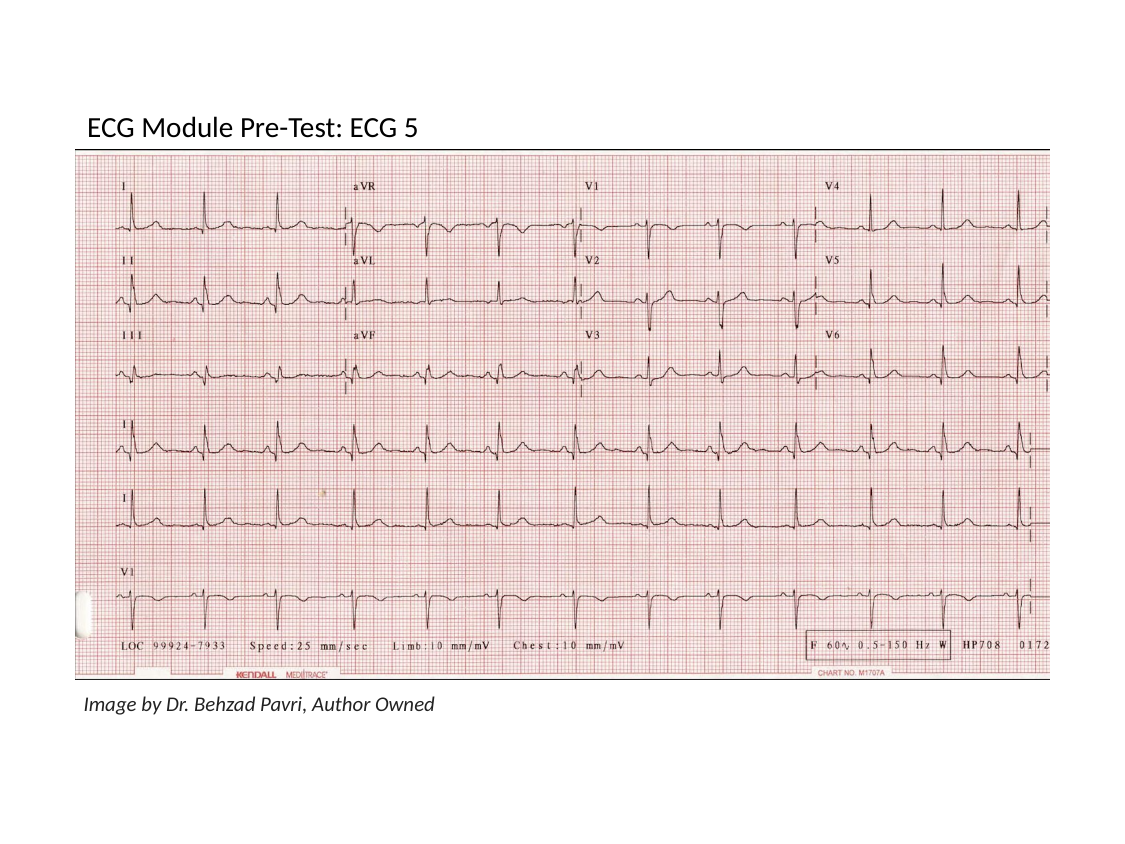

ECG Module Pre-Test: ECG 5
Image by Dr. Behzad Pavri, Author Owned

## Slide 9
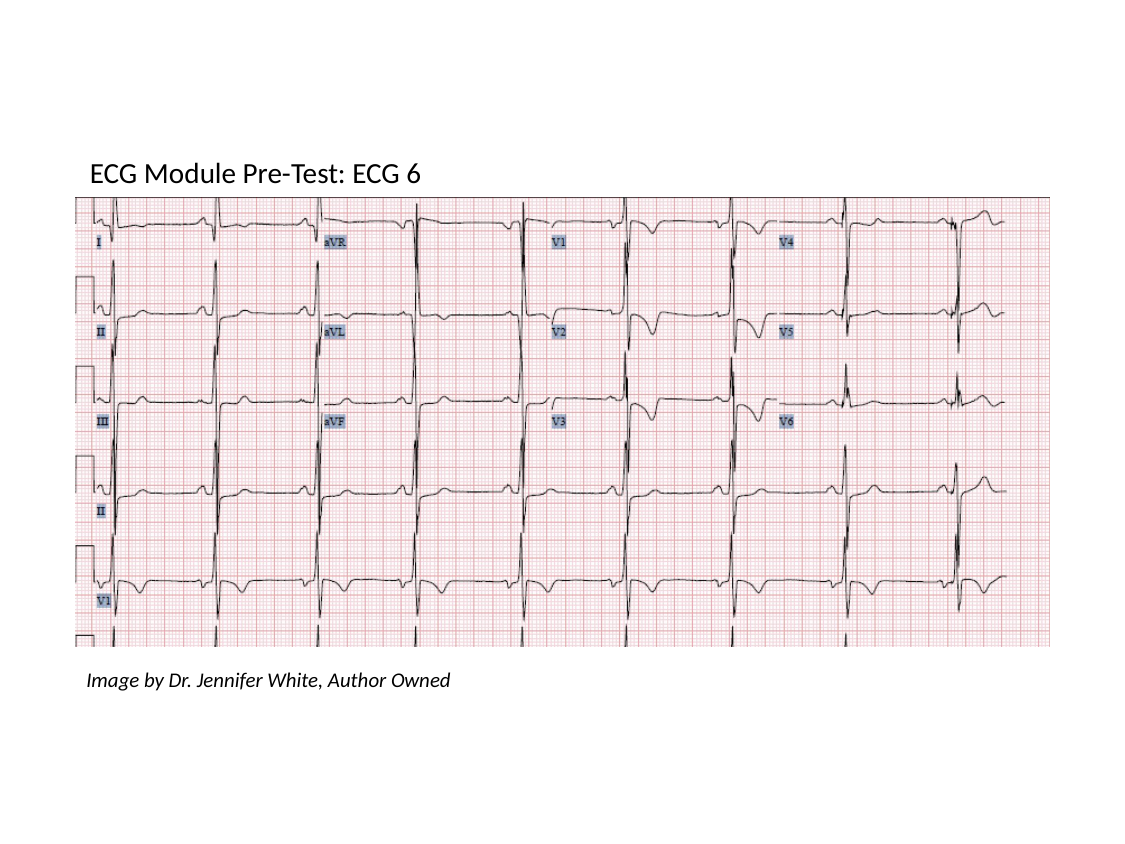

ECG Module Pre-Test: ECG 6
Image by Dr. Jennifer White, Author Owned

## Slide 10
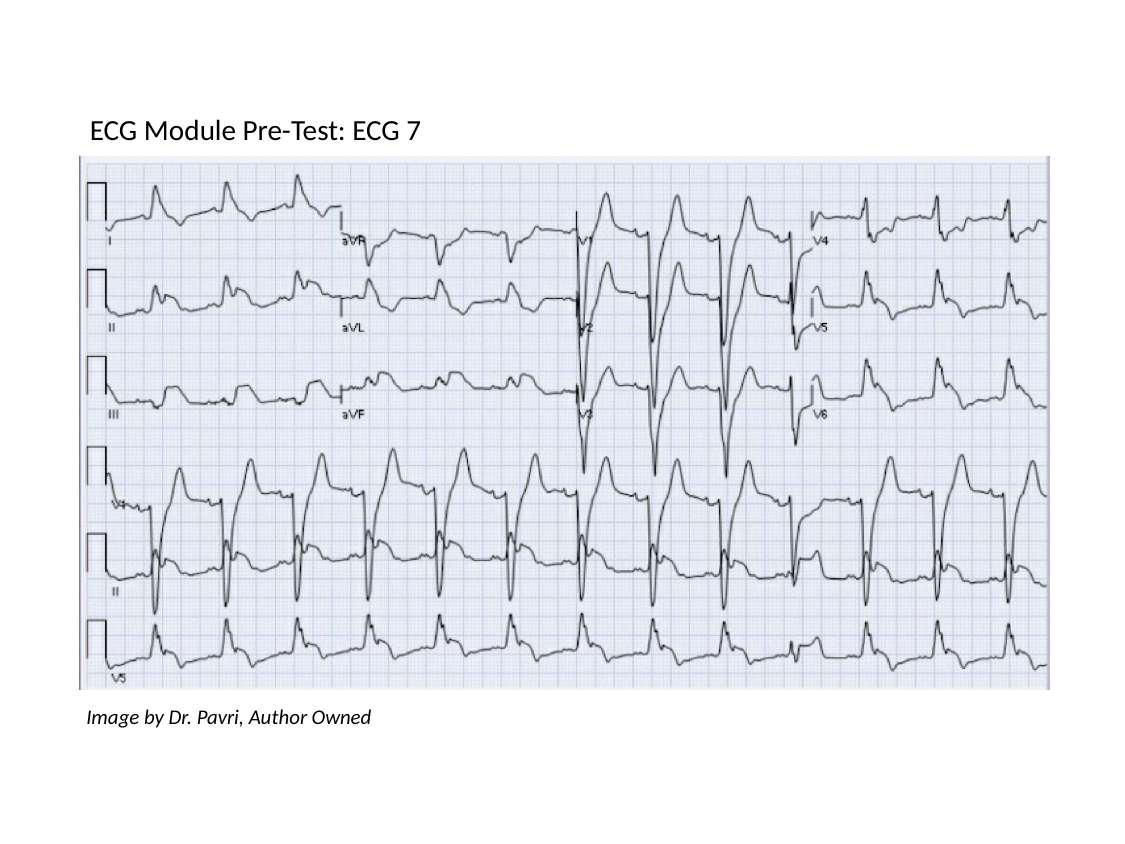

ECG Module Pre-Test: ECG 7
Image by Dr. Pavri, Author Owned

## Slide 11
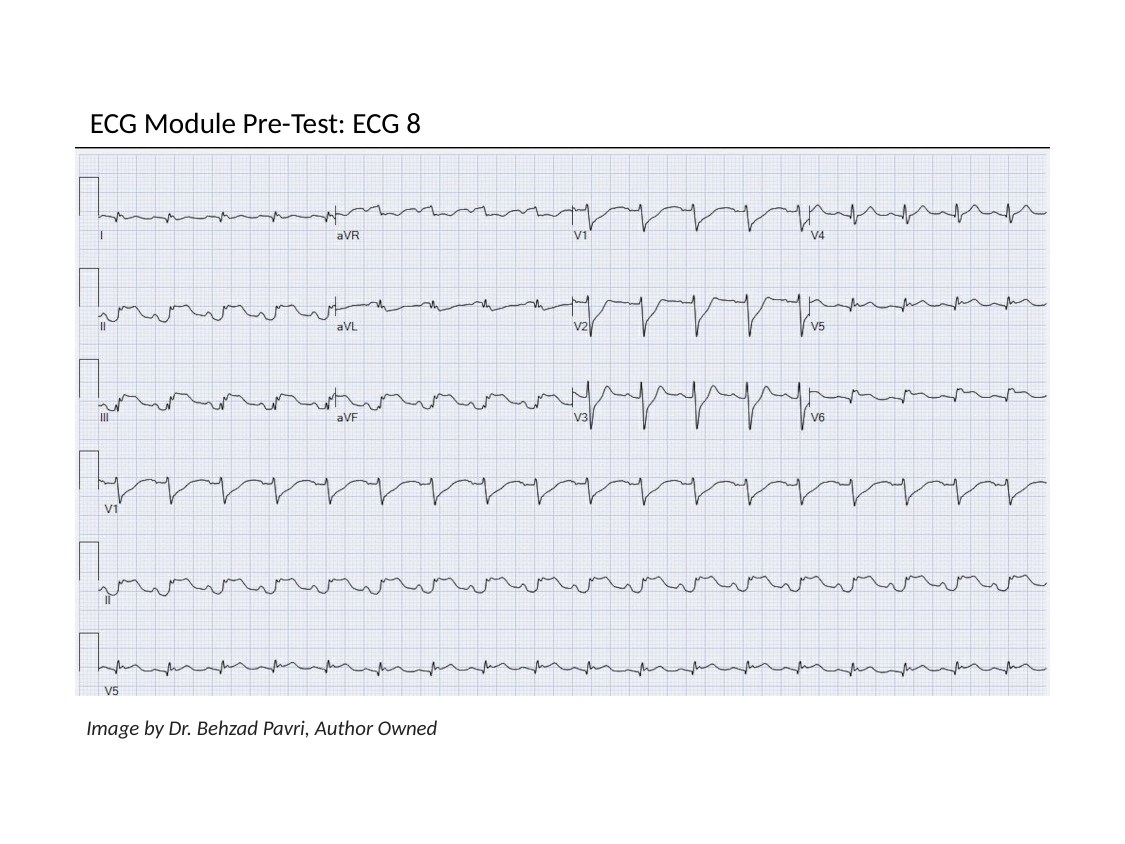

ECG Module Pre-Test: ECG 8
Image by Dr. Behzad Pavri, Author Owned

## Slide 12
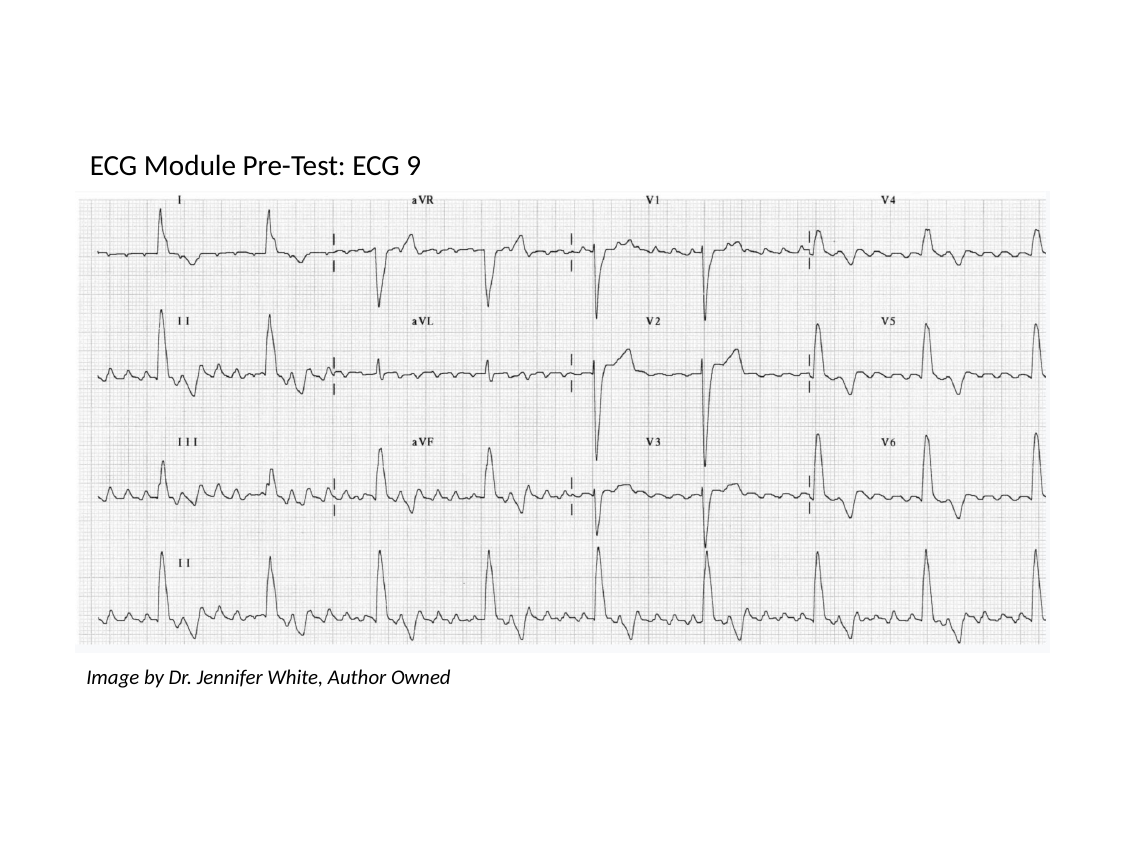

ECG Module Pre-Test: ECG 9
Image by Dr. Jennifer White, Author Owned

## Slide 13
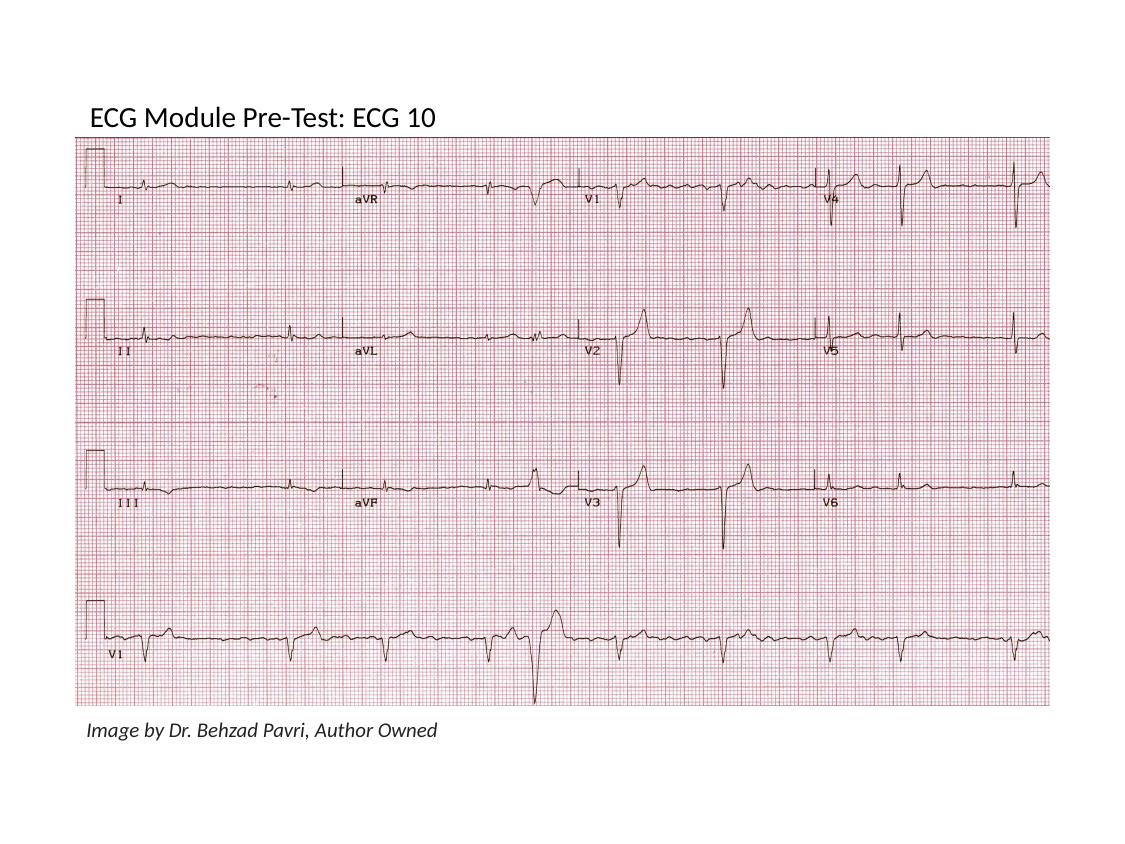

ECG Module Pre-Test: ECG 10
Image by Dr. Behzad Pavri, Author Owned

## Slide 14
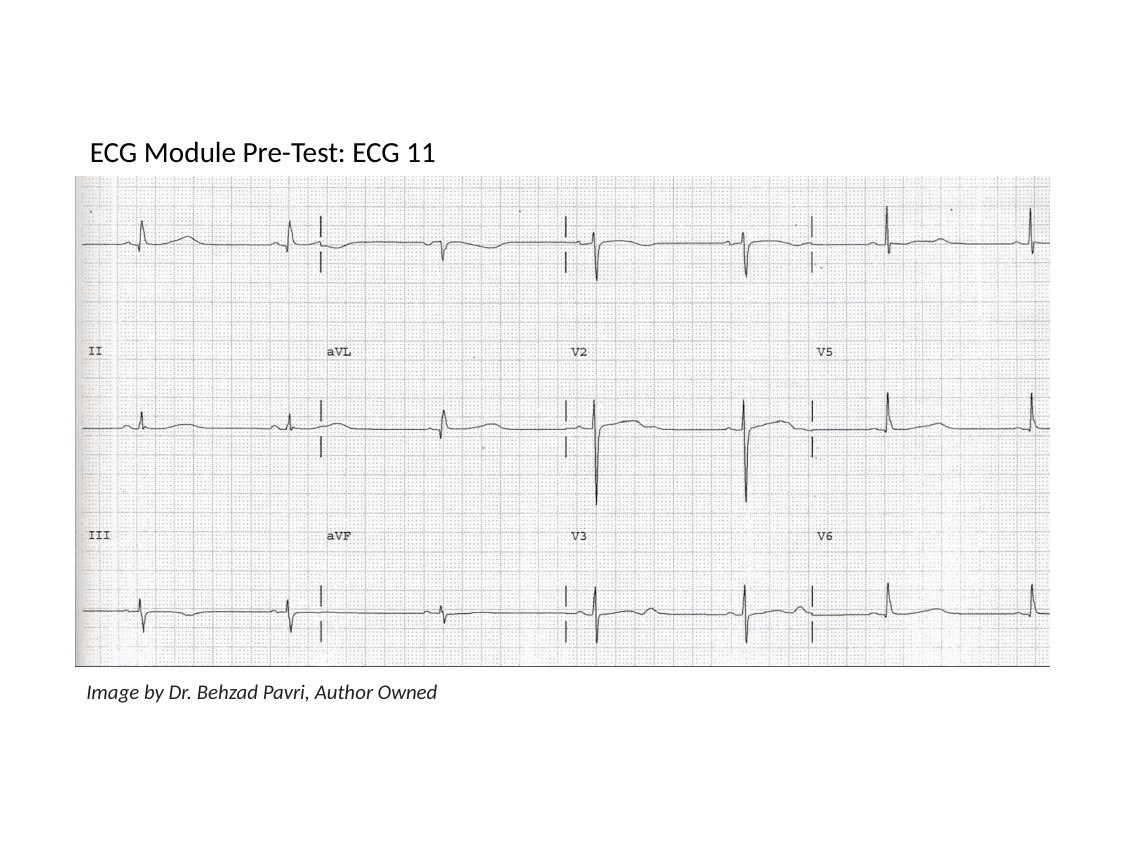

ECG Module Pre-Test: ECG 11
Image by Dr. Behzad Pavri, Author Owned

## Slide 15
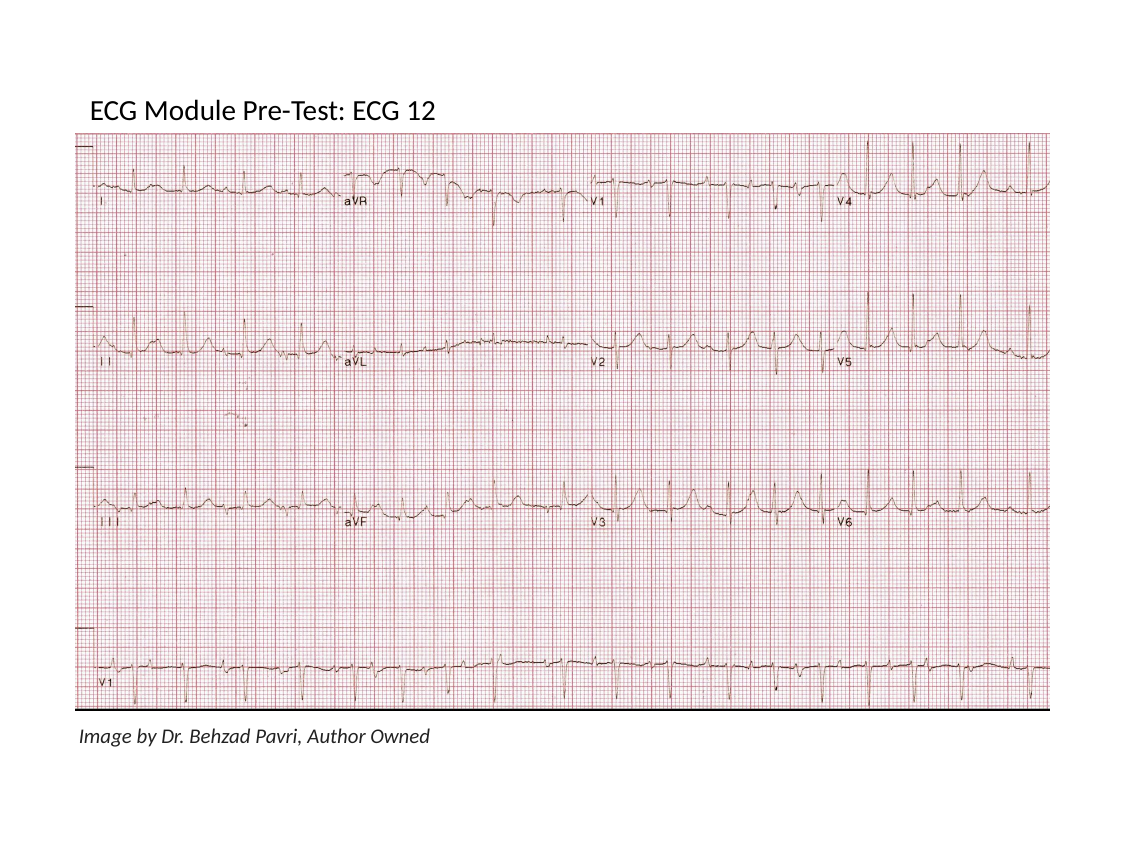

ECG Module Pre-Test: ECG 12
Image by Dr. Behzad Pavri, Author Owned

## Slide 16
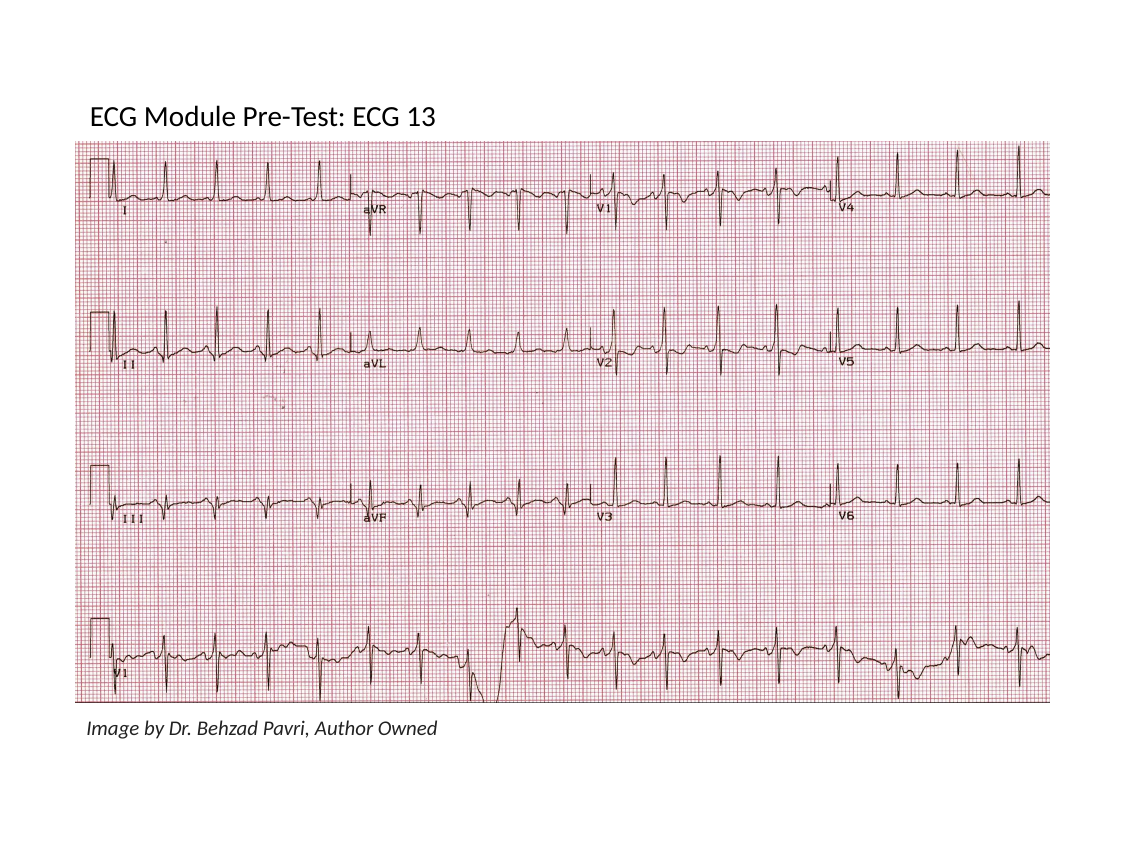

ECG Module Pre-Test: ECG 13
Image by Dr. Behzad Pavri, Author Owned

## Slide 17
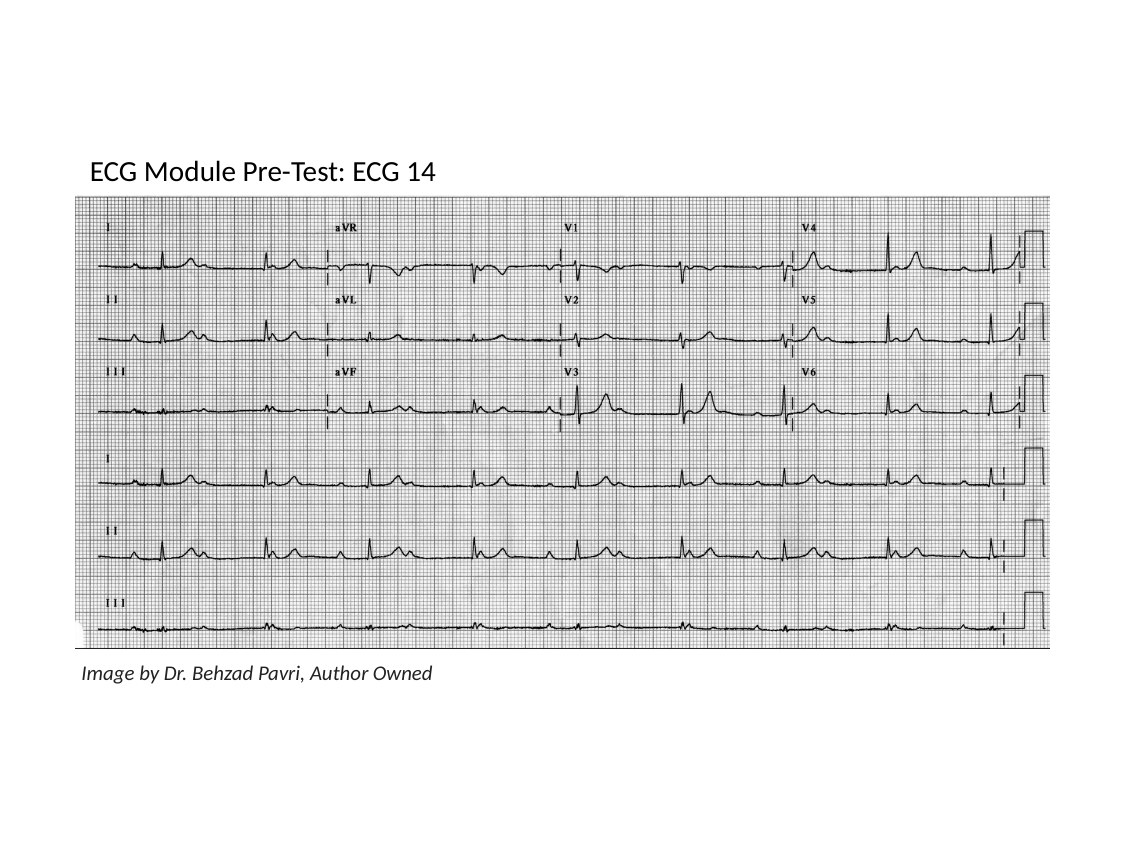

ECG Module Pre-Test: ECG 14
Image by Dr. Behzad Pavri, Author Owned

## Slide 18
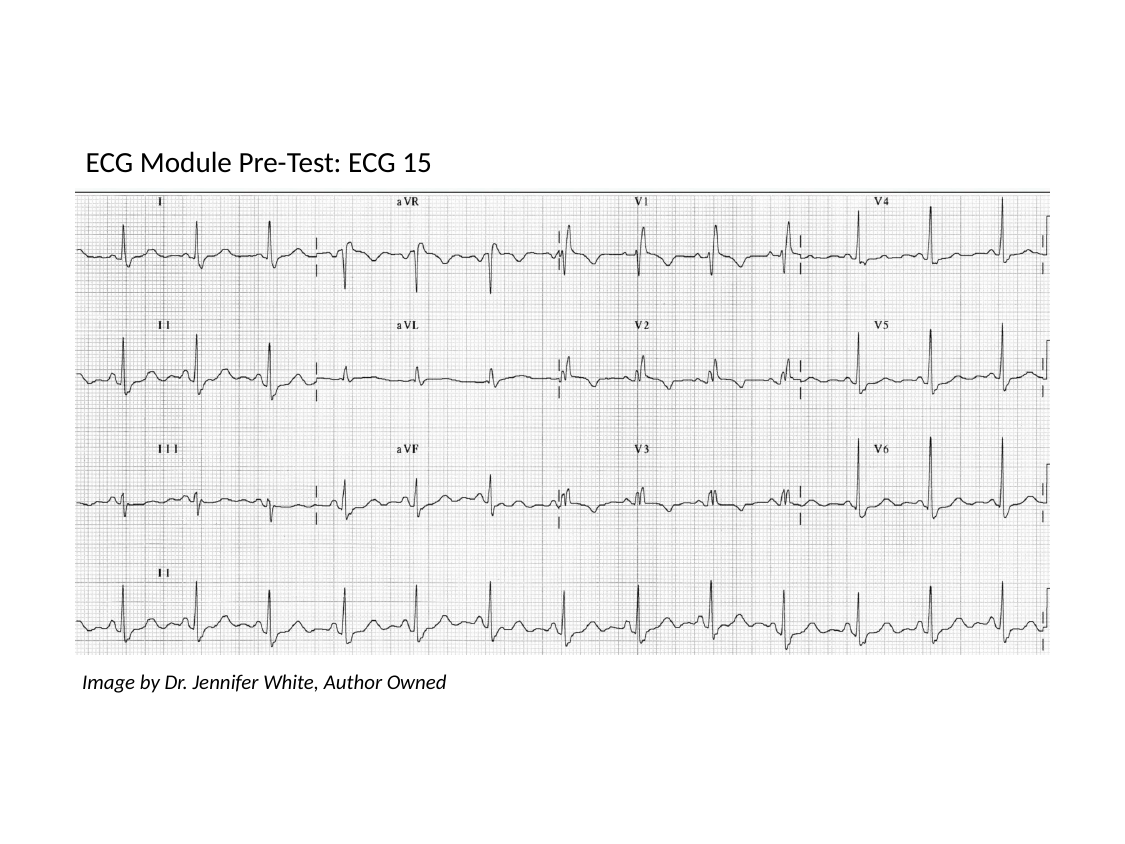

ECG Module Pre-Test: ECG 15
Image by Dr. Jennifer White, Author Owned
