# Supplementary material for: A Novel Module Based Method of Teaching Electrocardiogram Interpretation for Emergency Medicine Residents
Source: J Educ Teach Emerg Med. 2022 Oct 15;7(4):SG15–60. doi: 10.21980/J8Z06J (PMC10332672; doi:10.21980/J8Z06J)
Supplement: Supplementary file 3 [file JETem-7-4-SG15-AppendixB.docx]

Appendix B:

Pre-Test Answers

1. Incomplete Right bundle branch block (RBBB), with probable left posterior fascicular block
2. Non-ST elevation myocardial infarct (NSTEMI)
3. Brugada
4. Mobitz Type I
5. Normal sinus rhythm
6. Hypertrophic cardiomyopathy
7. Left bundle branch block (LBBB) with ST-Elevation myocardial infarct (STEMI)
8. Acute STEMI, inferoposterior
9. Atrial flutter (atypical) with intraventricular conduction delay (also accepting LBBB)
10. Atrial fibrillation with a slow ventricular response and 1 PVC
11. Marked sinus bradycardia with a long QT
12. Multifocal atrial tachycardia
13. Sinus tachycardia with Wolff Parkinson White
14. Complete heart block with narrow escape
15. RBBB
