# Supplementary material for: A Novel Module Based Method of Teaching Electrocardiogram Interpretation for Emergency Medicine Residents
Source: J Educ Teach Emerg Med. 2022 Oct 15;7(4):SG15–60. doi: 10.21980/J8Z06J (PMC10332672; doi:10.21980/J8Z06J)
Supplement: Supplementary file 4 — https://rise.articulate.com/share/YN7pQLkc3HJeTHSnLqCLK-RRQLk8j3Uj [file JETem-7-4-SG15-AppendixC.docx]

Appendix C:

Link to Online Module

[
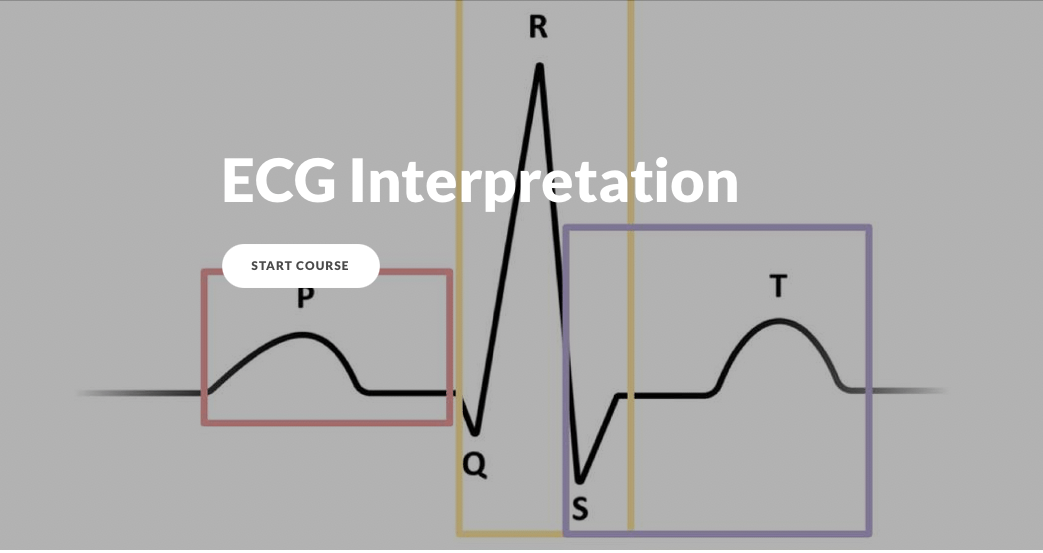
](https://rise.articulate.com/share/YN7pQLkc3HJeTHSnLqCLK-RRQLk8j3Uj)

<https://rise.articulate.com/share/YN7pQLkc3HJeTHSnLqCLK-RRQLk8j3Uj>
