# Supplementary material for: A Novel Module Based Method of Teaching Electrocardiogram Interpretation for Emergency Medicine Residents
Source: J Educ Teach Emerg Med. 2022 Oct 15;7(4):SG15–60. doi: 10.21980/J8Z06J (PMC10332672; doi:10.21980/J8Z06J)
Supplement: Supplementary file 6 [file JETem-7-4-SG15-AppendixD2.pptx]

## Slide 1
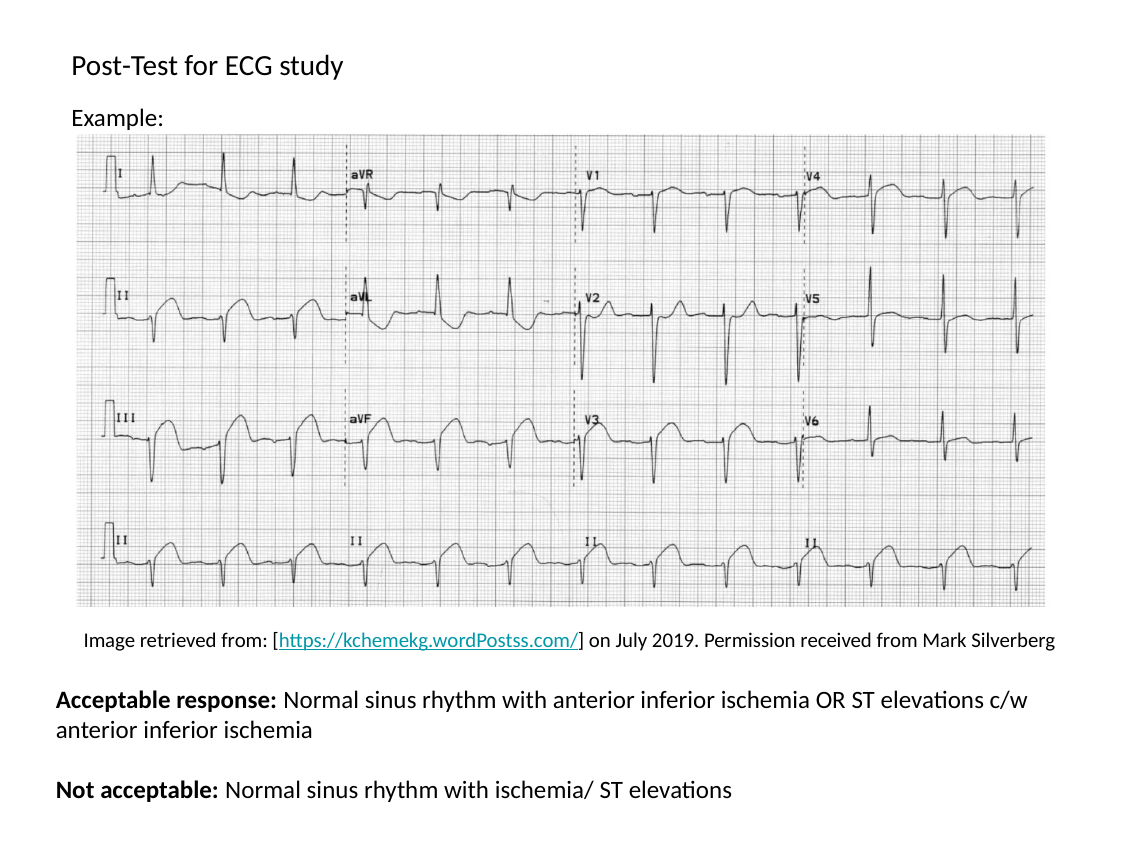

Post-Test for ECG study
Example:
Image retrieved from: [https://kchemekg.wordPostss.com/] on July 2019. Permission received from Mark Silverberg
Acceptable response: Normal sinus rhythm with anterior inferior ischemia OR ST elevations c/w anterior inferior ischemia
Not acceptable: Normal sinus rhythm with ischemia/ ST elevations

## Slide 2
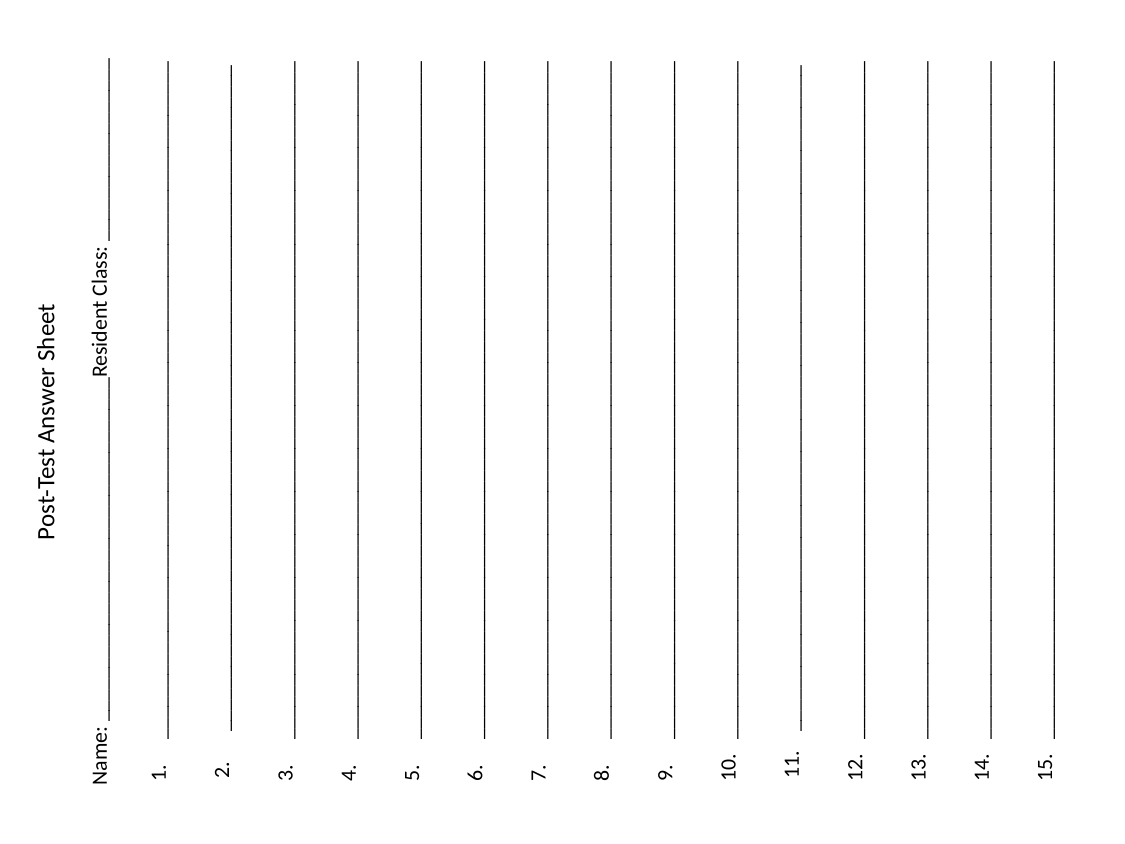

Post-Test Answer Sheet
Name: ________________________________Resident Class: _________________
_______________________________________________________________
 ______________________________________________________________
_______________________________________________________________
_______________________________________________________________
_______________________________________________________________
_______________________________________________________________
_______________________________________________________________
_______________________________________________________________
_______________________________________________________________
_______________________________________________________________
 ______________________________________________________________
_______________________________________________________________
_______________________________________________________________
_______________________________________________________________
_______________________________________________________________

## Slide 3
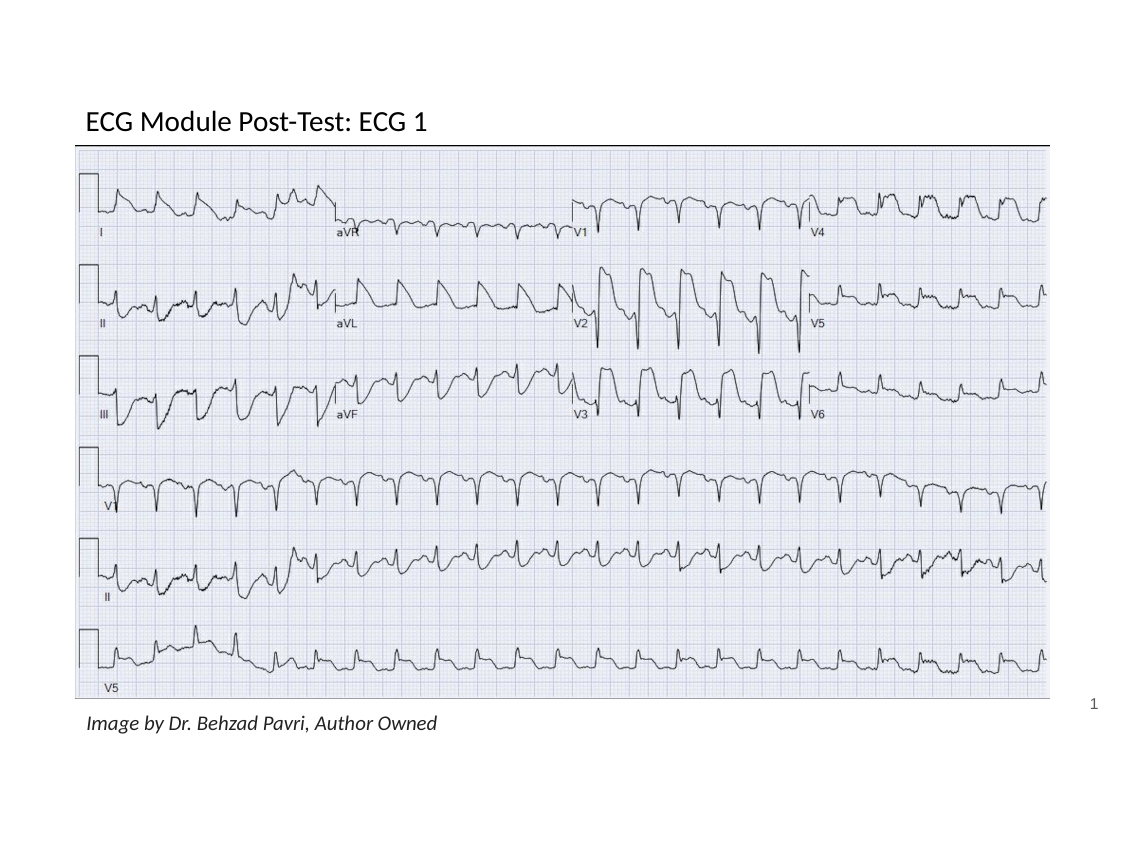

ECG Module Post-Test: ECG 1
1
Image by Dr. Behzad Pavri, Author Owned

## Slide 4
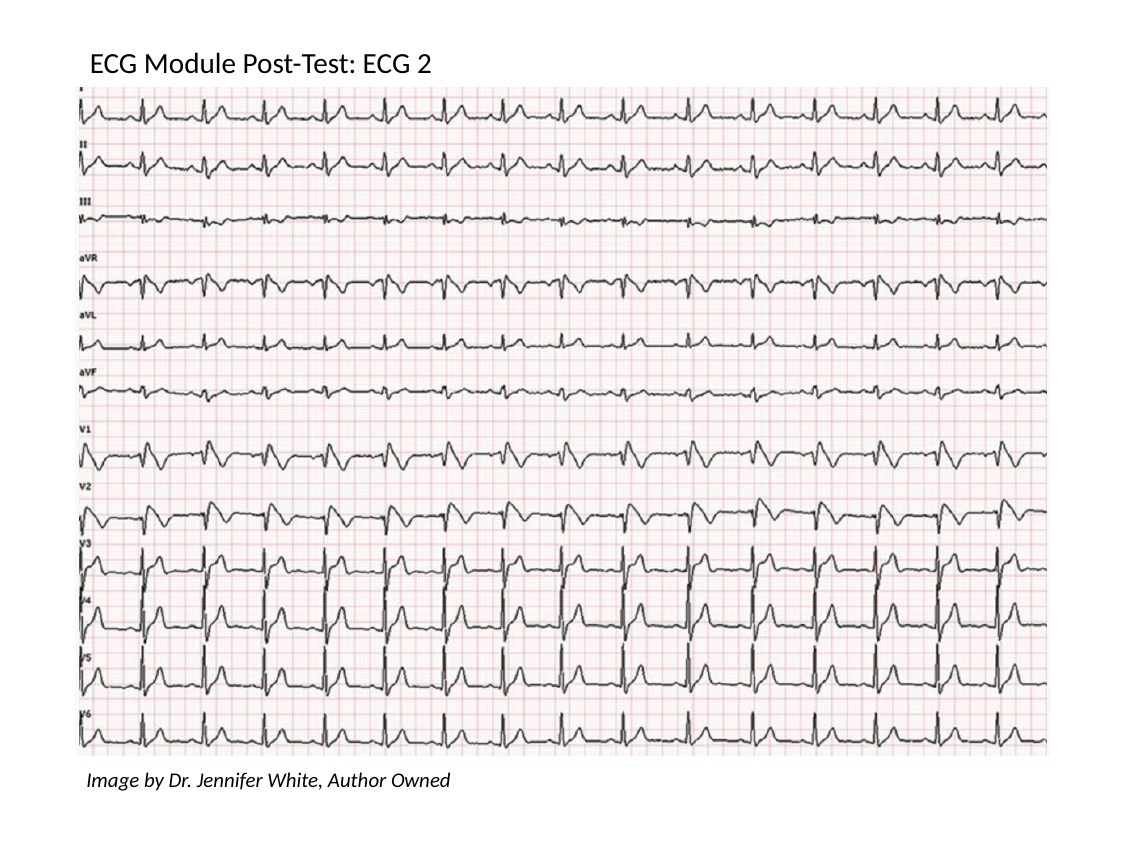

ECG Module Post-Test: ECG 2
Image by Dr. Jennifer White, Author Owned

## Slide 5
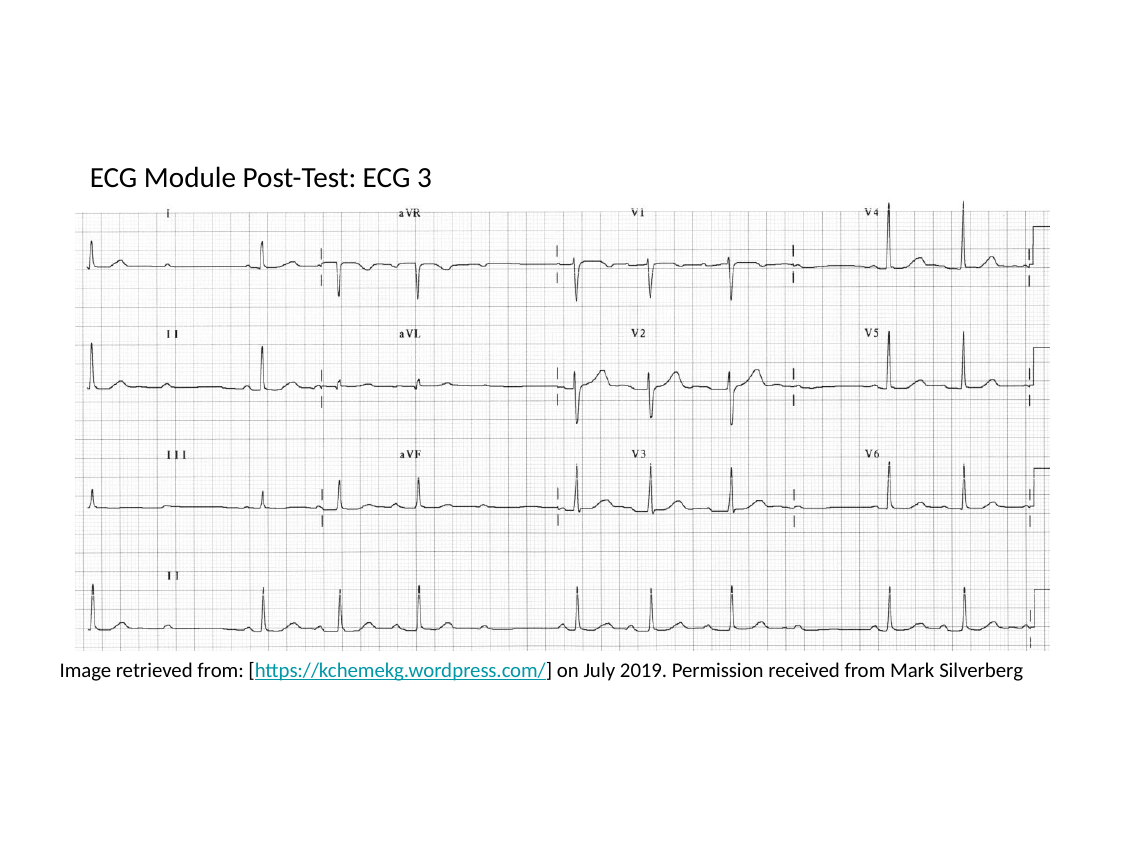

ECG Module Post-Test: ECG 3
Image retrieved from: [https://kchemekg.wordpress.com/] on July 2019. Permission received from Mark Silverberg

## Slide 6
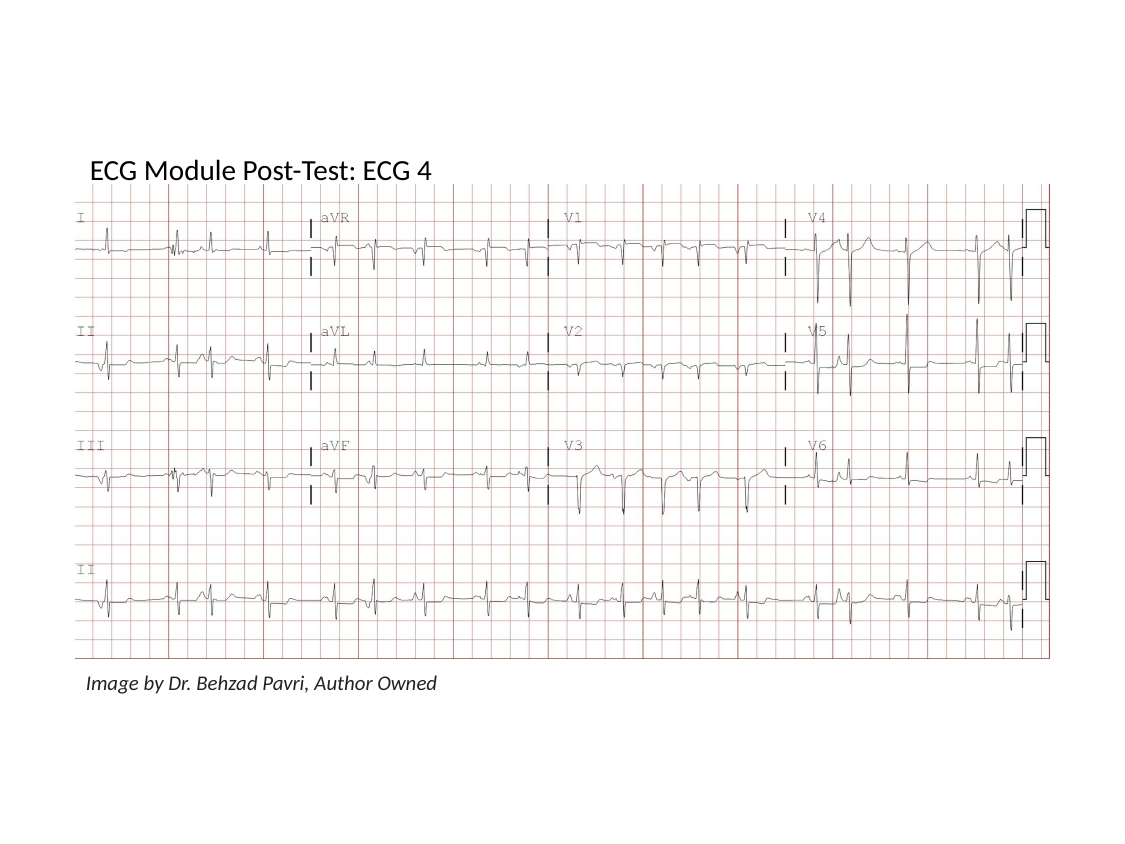

ECG Module Post-Test: ECG 4
Image by Dr. Behzad Pavri, Author Owned

## Slide 7
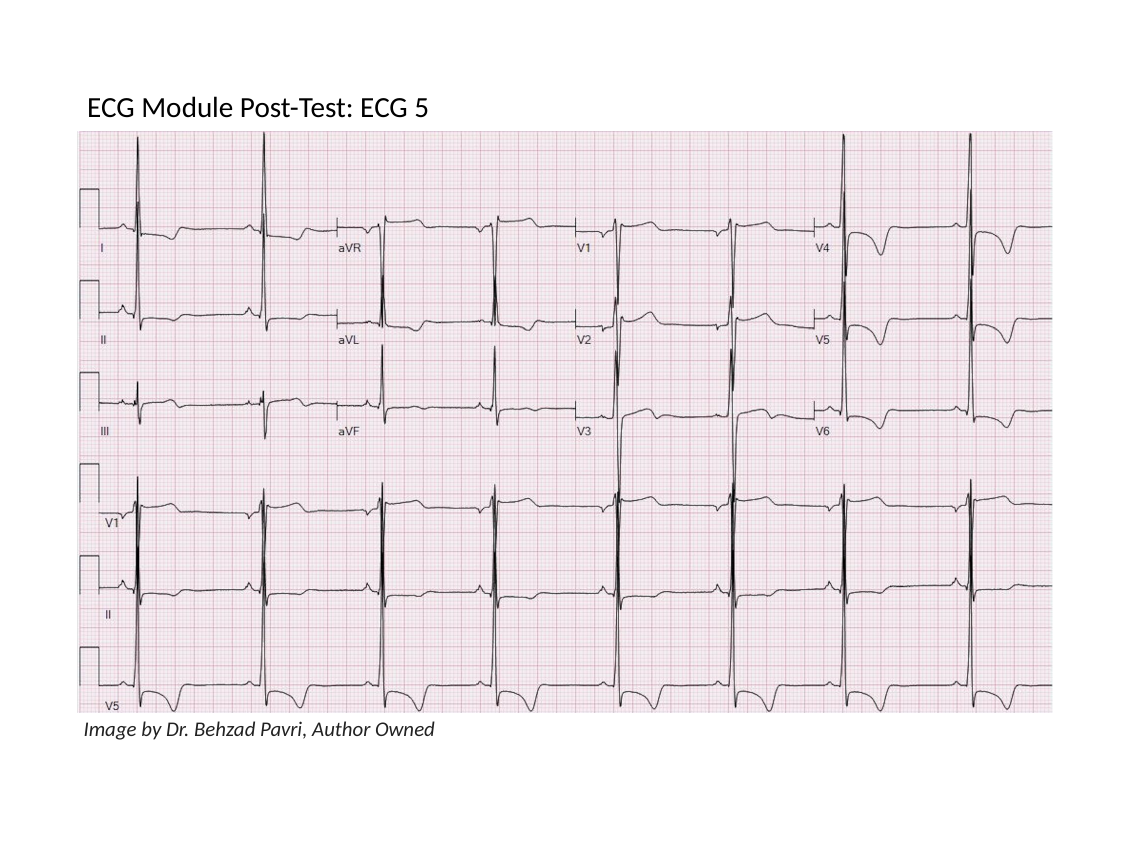

ECG Module Post-Test: ECG 5
Image by Dr. Behzad Pavri, Author Owned

## Slide 8
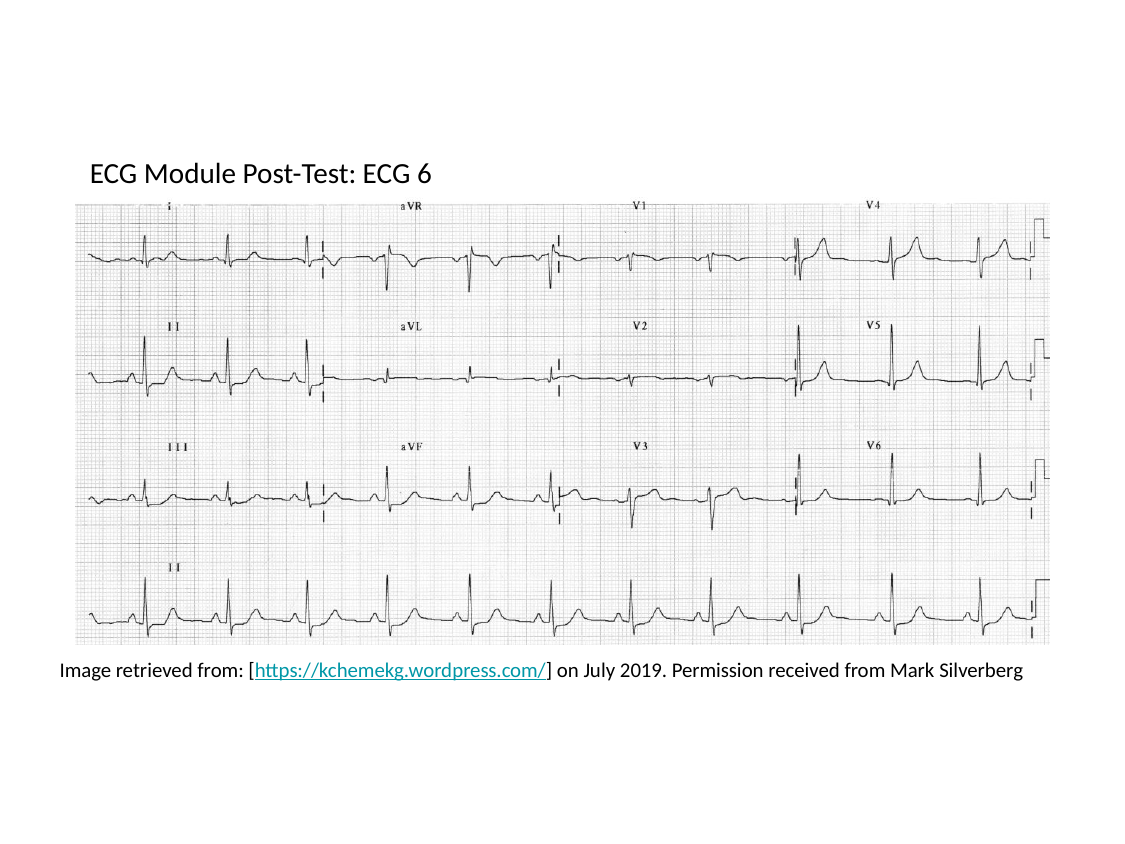

ECG Module Post-Test: ECG 6
Image retrieved from: [https://kchemekg.wordpress.com/] on July 2019. Permission received from Mark Silverberg

## Slide 9
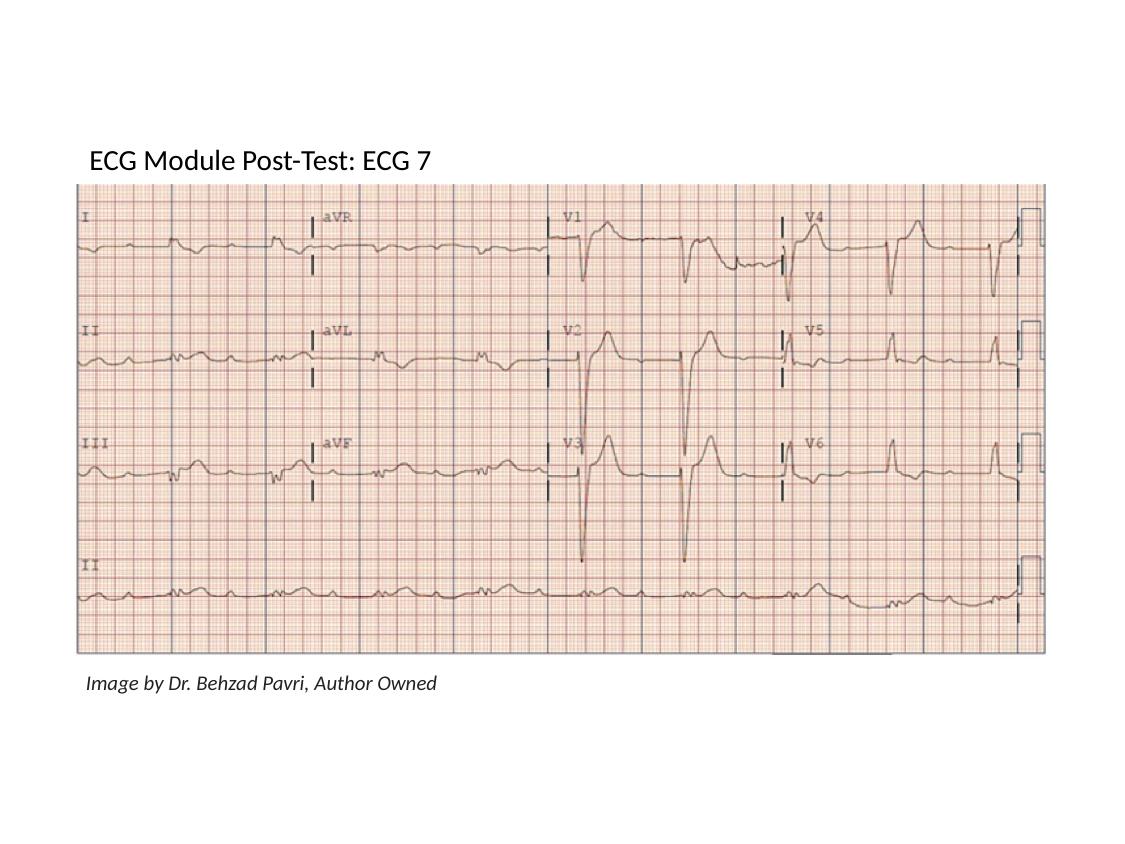

ECG Module Post-Test: ECG 7
Image by Dr. Behzad Pavri, Author Owned

## Slide 10
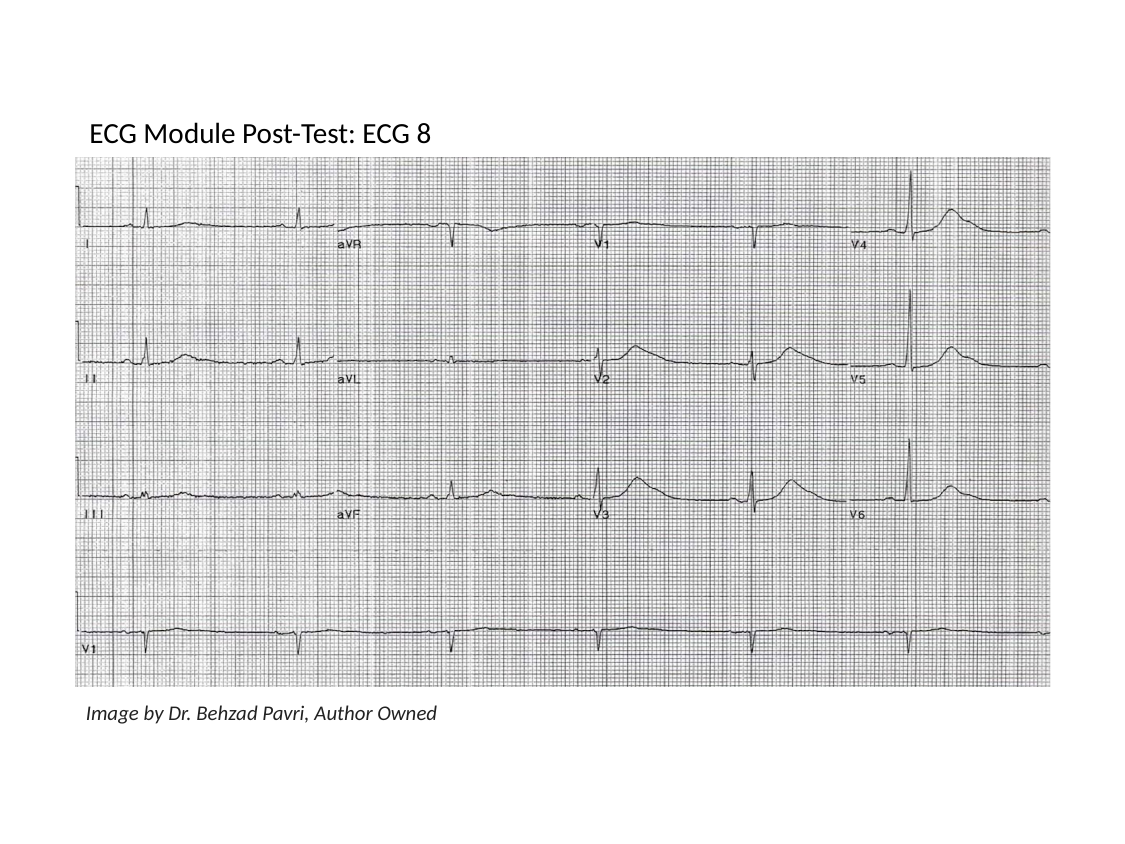

ECG Module Post-Test: ECG 8
Image by Dr. Behzad Pavri, Author Owned

## Slide 11
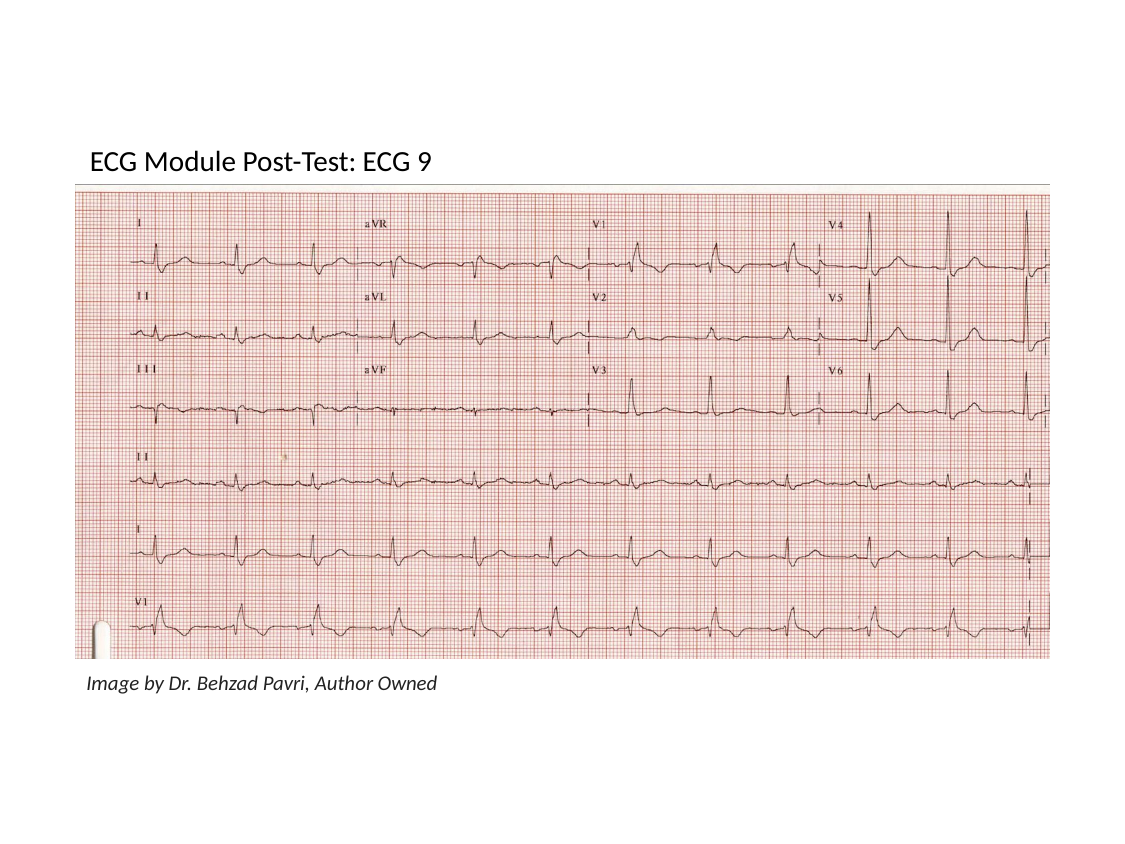

ECG Module Post-Test: ECG 9
Image by Dr. Behzad Pavri, Author Owned

## Slide 12
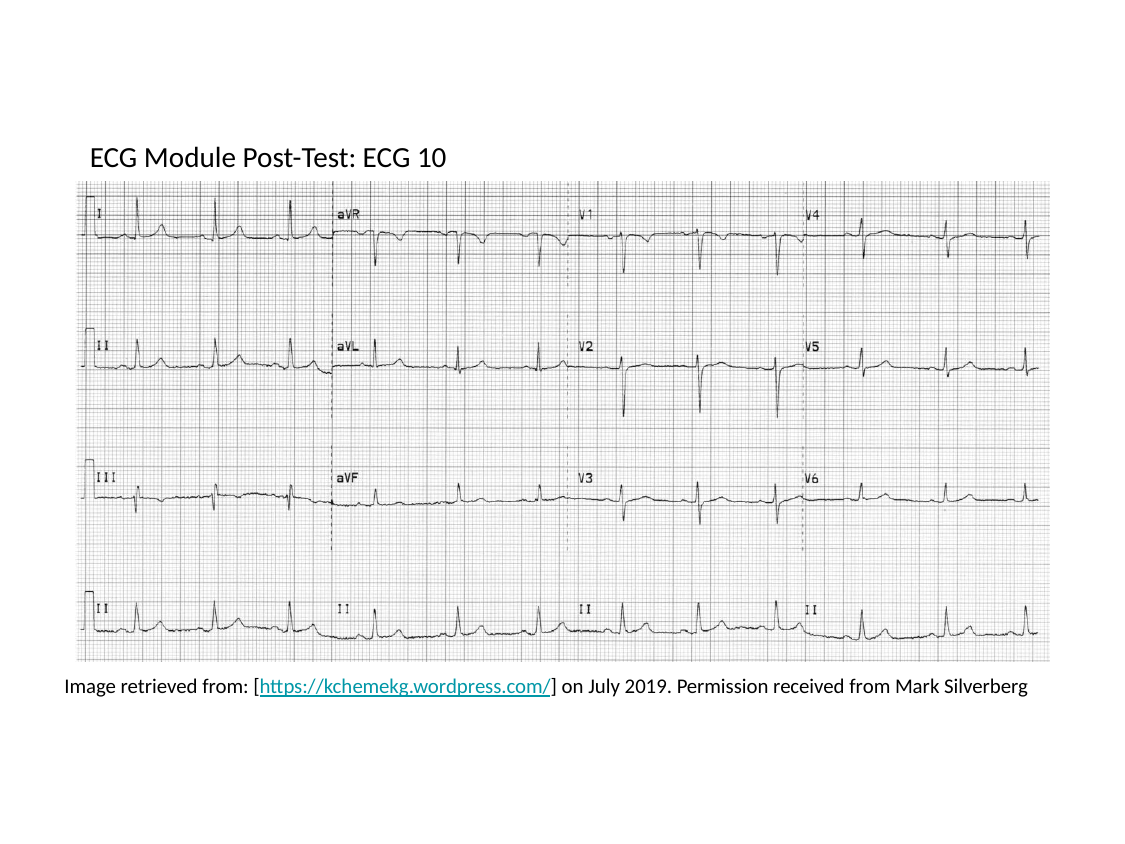

ECG Module Post-Test: ECG 10
Image retrieved from: [https://kchemekg.wordpress.com/] on July 2019. Permission received from Mark Silverberg

## Slide 13
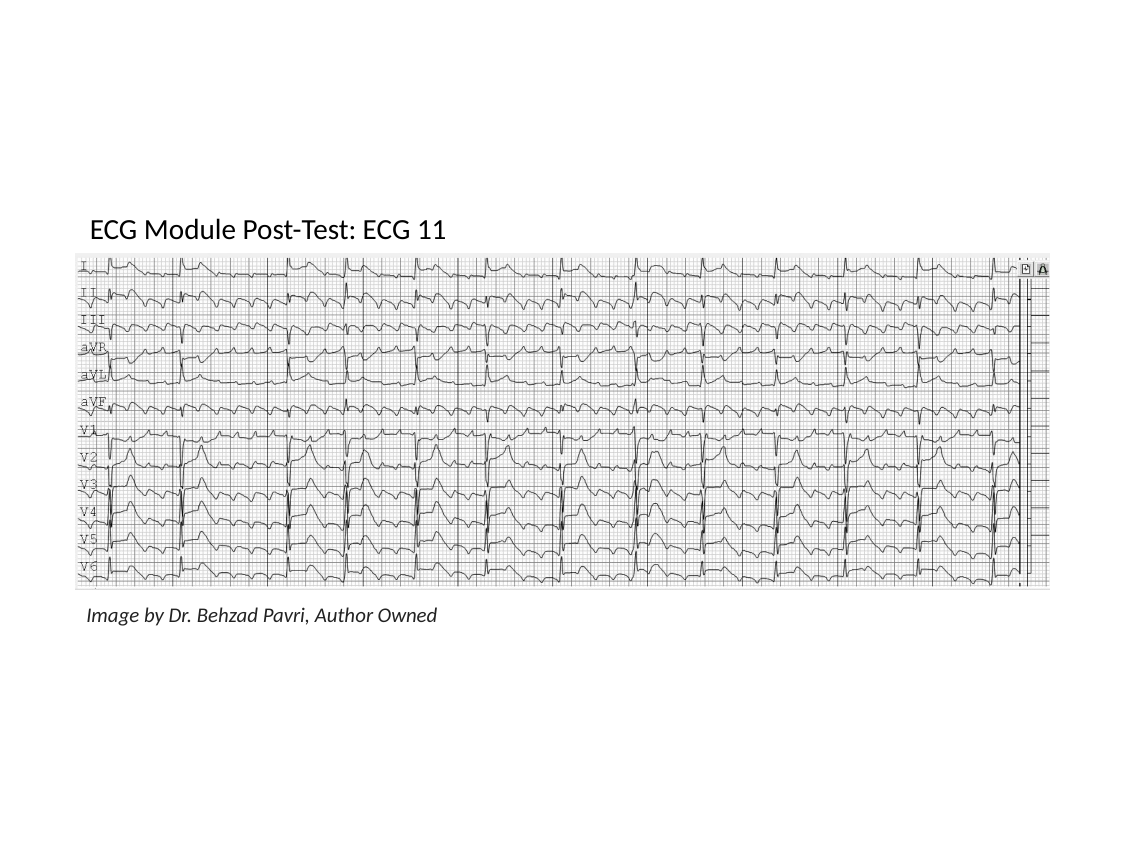

ECG Module Post-Test: ECG 11
Image by Dr. Behzad Pavri, Author Owned

## Slide 14
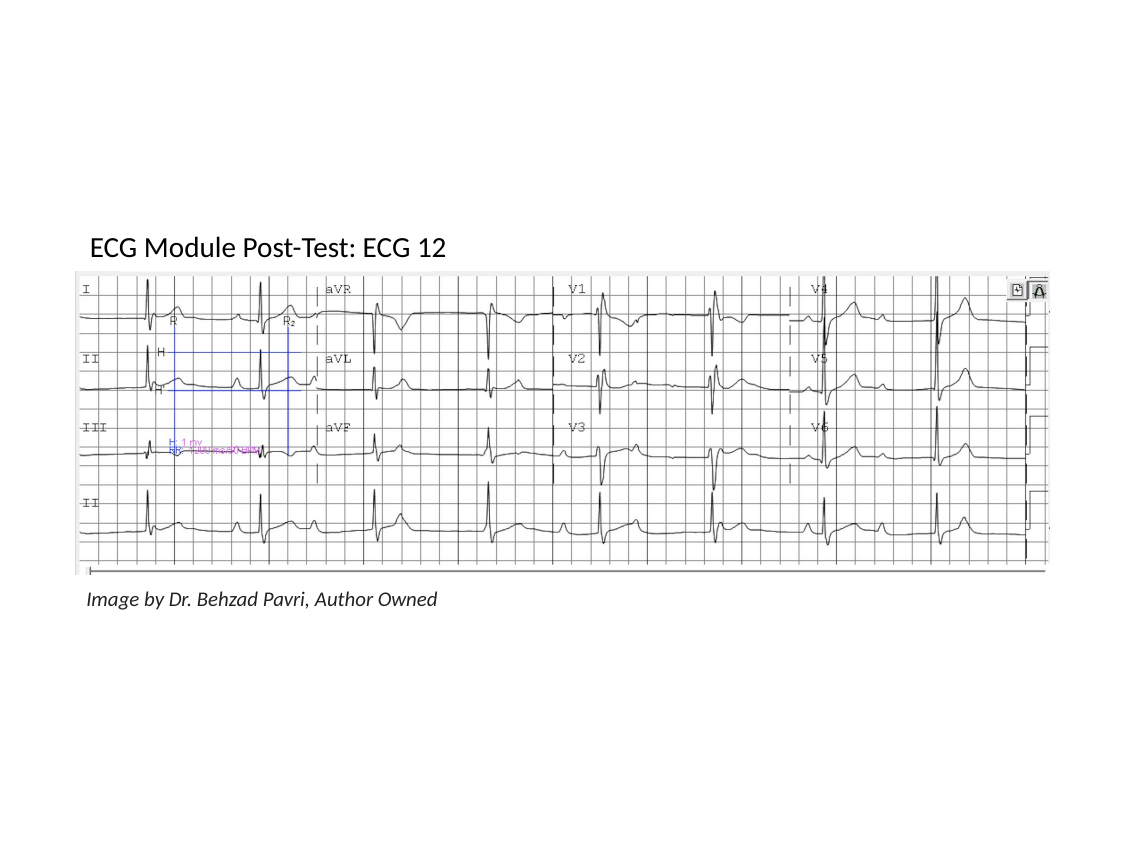

ECG Module Post-Test: ECG 12
Image by Dr. Behzad Pavri, Author Owned

## Slide 15
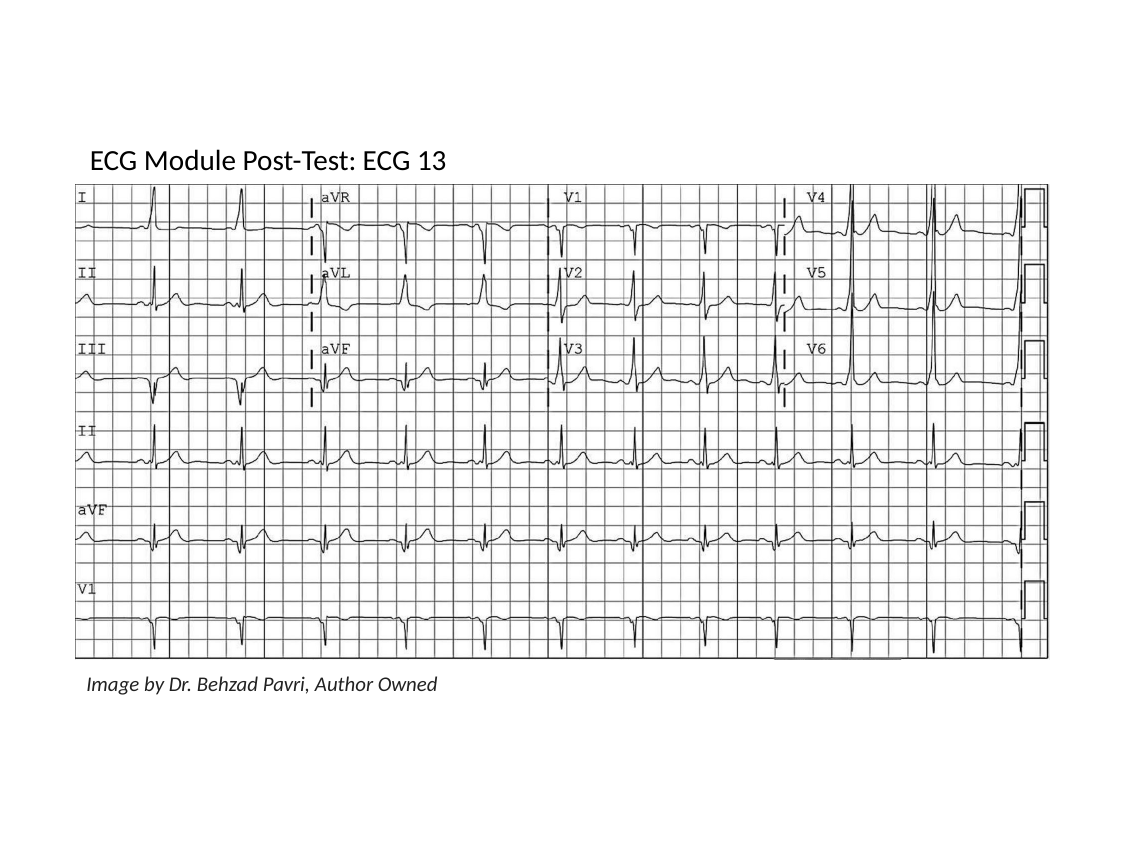

ECG Module Post-Test: ECG 13
Image by Dr. Behzad Pavri, Author Owned

## Slide 16
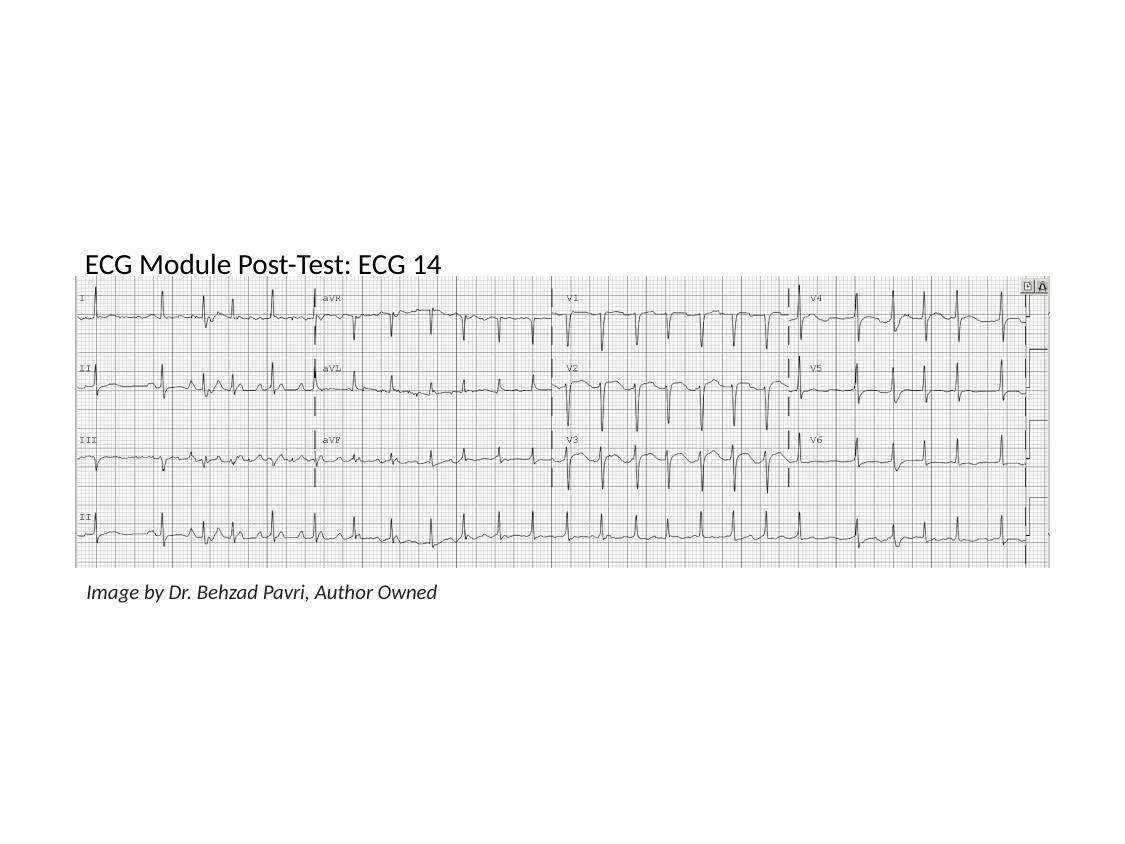

ECG Module Post-Test: ECG 14
Image by Dr. Behzad Pavri, Author Owned

## Slide 17
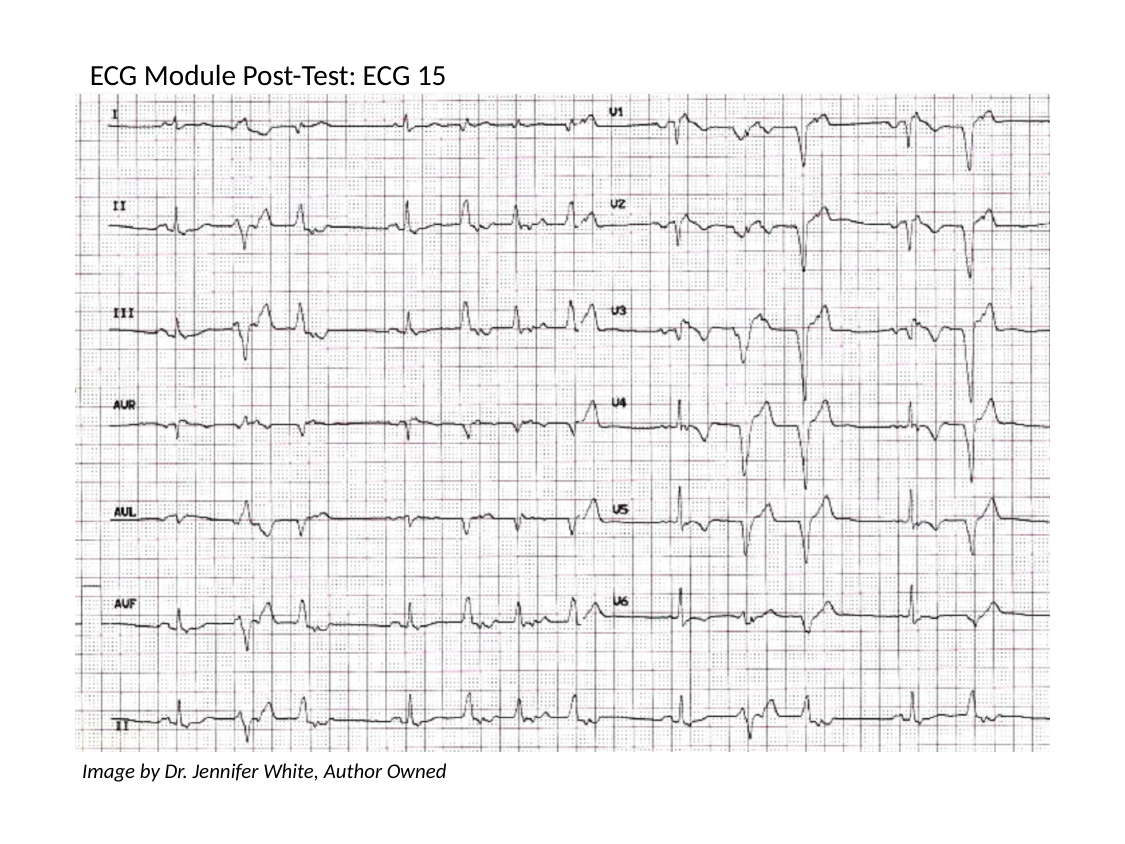

ECG Module Post-Test: ECG 15
Image by Dr. Jennifer White, Author Owned

## Slide 18
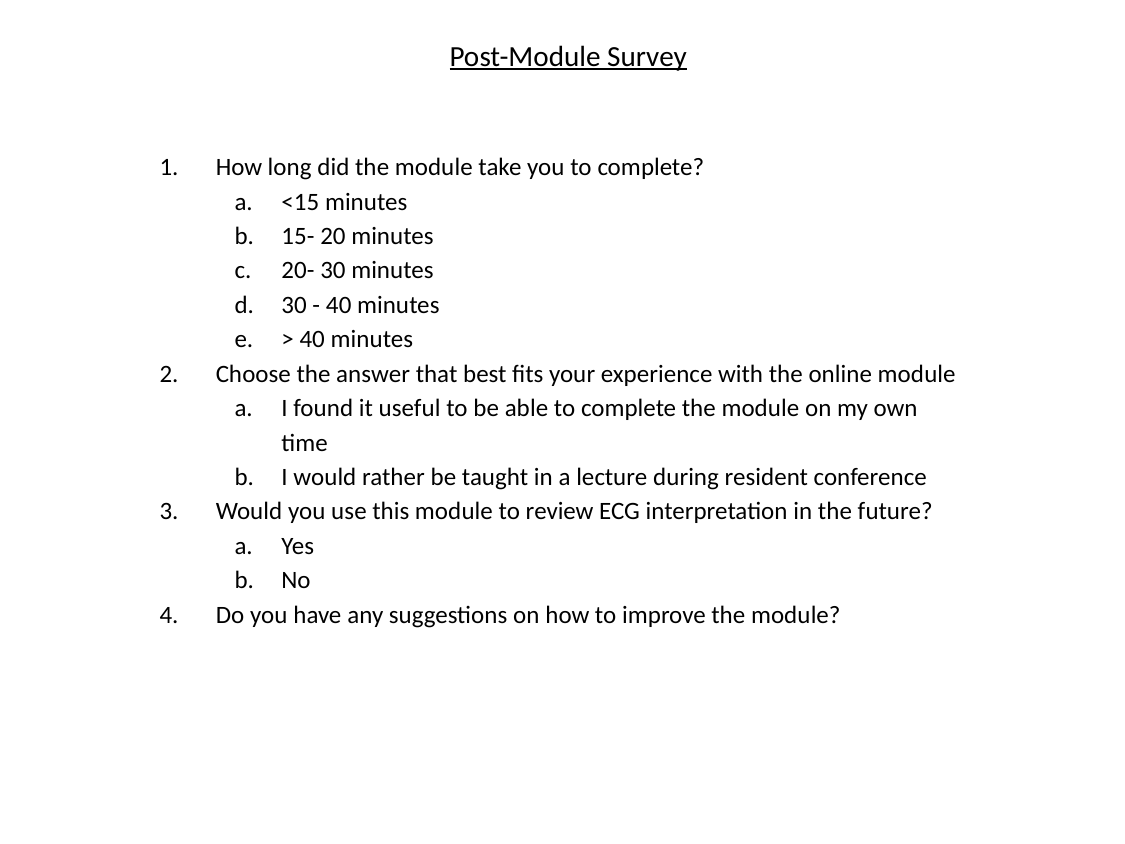

Post-Module Survey
How long did the module take you to complete?
<15 minutes
15- 20 minutes
20- 30 minutes
30 - 40 minutes
> 40 minutes
Choose the answer that best fits your experience with the online module
I found it useful to be able to complete the module on my own time
I would rather be taught in a lecture during resident conference
Would you use this module to review ECG interpretation in the future?
Yes
No
Do you have any suggestions on how to improve the module?

## Slide 19
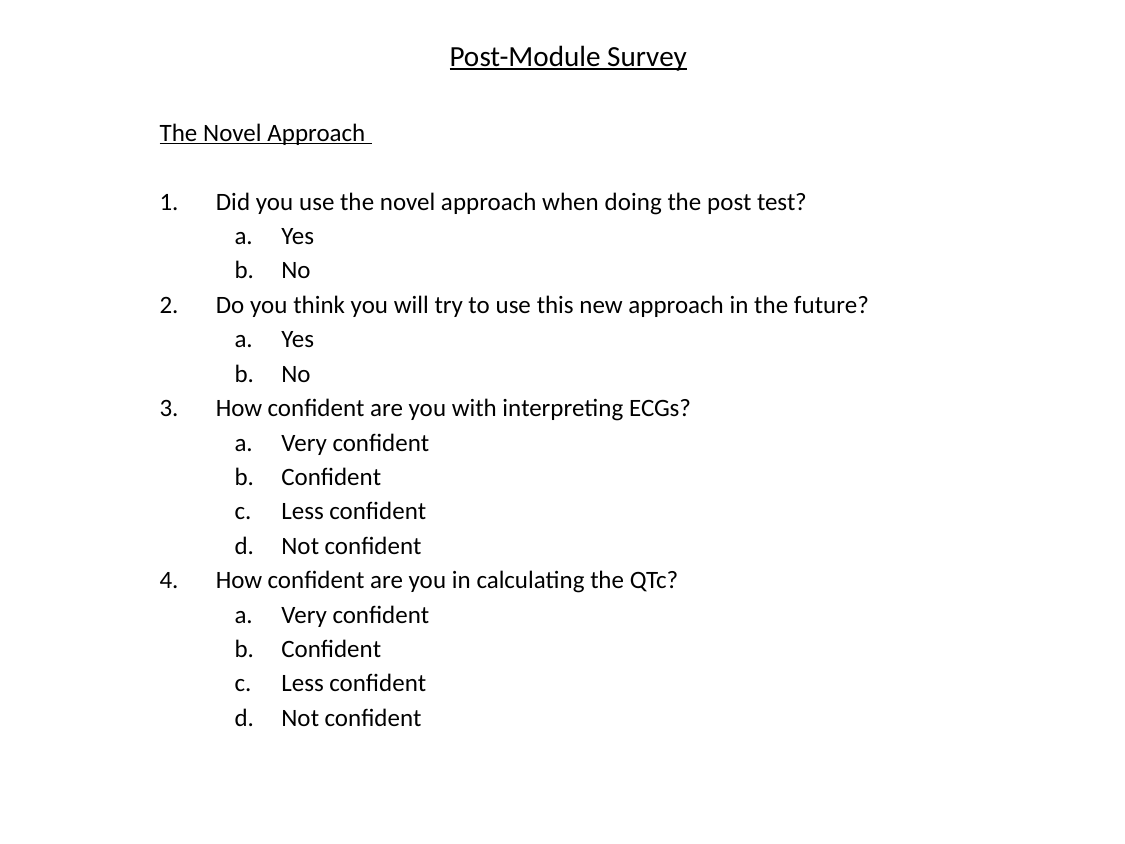

Post-Module Survey
The Novel Approach
Did you use the novel approach when doing the post test?
Yes
No
Do you think you will try to use this new approach in the future?
Yes
No
How confident are you with interpreting ECGs?
Very confident
Confident
Less confident
Not confident
How confident are you in calculating the QTc?
Very confident
Confident
Less confident
Not confident
