# Supplementary material for: A Novel Module Based Method of Teaching Electrocardiogram Interpretation for Emergency Medicine Residents
Source: J Educ Teach Emerg Med. 2022 Oct 15;7(4):SG15–60. doi: 10.21980/J8Z06J (PMC10332672; doi:10.21980/J8Z06J)
Supplement: Supplementary file 7 [file JETem-7-4-SG15-AppendixE.docx]

Appendix E:

Post-Test Answers

1. Sinus tachycardia with proximal LAD STEMI
2. Brugada
3. Second degree AV block type 1
4. Multifocal atrial tachycardia
5. Hypertrophic cardiomyopathy
6. NSTEMI
7. LBBB with STEMI
8. Sinus bradycardia with Long QT
9. Right bundle branch block
10. Normal sinus
11. Atrial flutter
12. Complete heart block with RBBB escape
13. Wolff Parkinson White
14. Atrial fibrillation (onset caught)
15. Arrhythmogenic right ventricular cardiomyopathy with frequent PVCs
